# Digital methods to quantify sensor output uncertainty in real time

**DOI:** 10.1038/s44172-026-00679-4

**Published:** 2026-05-13

**Authors:** Orestis Kaparounakis, Phillip Stanley-Marbell

**Affiliations:** 1 Physical Computation Laboratory, Department of Engineering, University of Cambridge, Cambridge, UK; 2 Signaloid, Cambridge, UK

**Keywords:** Computational science, Electrical and electronic engineering, Scientific data

## Abstract

Modern data-driven applications that make real-time decisions increasingly depend on advanced sensors which use pre-stored calibration data. In such applications, accurate characterization of sensor output uncertainty is important for reliable data interpretation. Here, we present a method for real-time on-device dynamic uncertainty quantification for sensor outputs which depend on pre-stored calibration data. We show how sensor calibration compensation equations (essential in advanced sensing systems) propagate uncertainties resulting from the quantization of calibration parameters to the sensor output. We use a low-cost thermal sensor as a motivating example and show these ideas are practical and possible on actual embedded sensor systems by prototyping them on two commercially-available uncertainty-tracking hardware platforms with average power dissipation 16.7 mW and 147.15 mW. These achieve 42.9× and 94.4× speedup compared to the equal-accuracy Monte Carlo computation (the status quo). We present a proof-of-usefulness edge-detection application over ten test scenes where accuracy and precision show average improvement by 4.97 and 40.25 percentage points, respectively, trading off sensitivity. Another application example examines four different calibration-data storage scenarios and compute that a 48% increase in memory yields 75% smaller uncertainty metrics over the baseline. Our method enables better decision-making in critical applications where sensor data reliability is paramount.

## Abstract (web summary)

Orestis Kaparounakis and colleagues report a real-time method to track uncertainty in sensor outputs caused by low-precision calibration data. This could help sensing systems make better use of measurements when uncertainty changes from case to case.

## Introduction

Cutting-edge realtime cyber-physical systems are increasingly data-driven and rely on ever more complex sensors^1–4^ whose functionality depends on pre-stored factory calibration data^5–10^. For such systems, the efficient and effective real-time operation hinges on the sensor output accuracy and characterized uncertainty. Yet, the conventional approach to sensing–control integration quantifies uncertainty per subsystem in an offline manner as opposed to a holistic and real-time approach. This work demonstrates an approach that enables real-time on-device dynamic quantification of the uncertainty which arises due to pre-stored calibration data.

Uncertainty about the true value of a quantity is either *aleatoric* or *epistemic*. Epistemic uncertainty is due to incomplete information. For example, a 20-digit estimate of *π* is epistemically uncertain beyond the 20th digit. Aleatoric uncertainty is due to inherent randomness and no amount of extra information reduces it—for example, the outcome of a fair coin toss. This work shows how epistemic uncertainty in the calibration data of sensors impacts the sensor output uncertainty and offers a practical solution compatible with resource-constrained sensing systems.

**Fig. 1:**
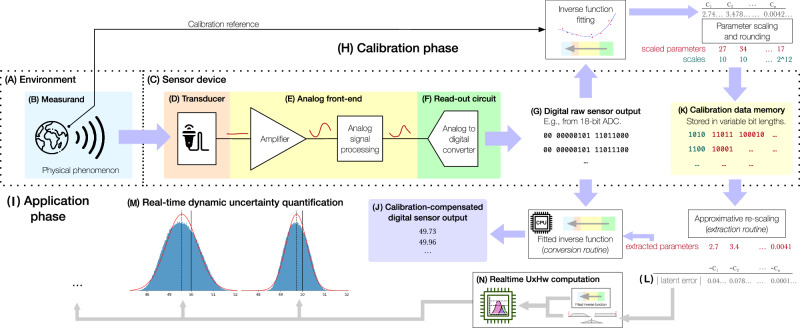
Simplified illustrative diagram for a calibration-compensated sensor. **A** The environment produces a physical phenomenon as an analog, potentially non-electrical, signal related to a quantity (**B**) we want to measure. **C** On the sensor device, a component picks up the signal and transduces it to an electrical signal (**D**). **E** An amplifier amplifies the electrical signal and analog circuits process it further. **F** An analog-to-digital converter turns measurements into digital values (**G**). These values do not have an obvious correspondence to the measurand. **H** The calibration phase uses reference values to fit a function (*conversion routine*) which applications (**I**) will use to compute measurand values (**J**) from the raw readings (**G**). The conversion routine uses parameters from the calibration phase (**H**) that the manufacturer pre-stored in the sensor device memory (**K**). Because these parameters are constrained in precision by finite digital memories, quantization errors (**L**) in the extracted parameters cause the sensor output of the conversion routines to contain significant amounts of epistemic uncertainty (**M**). **N** Conventional approaches can quantify this uncertainty in a static off-device offline approach: this work presents a method for dynamic on-device real-time uncertainty quantification for sensor outputs which depend on pre-stored calibration data.

Figure 1 shows a simplified overview of a calibration-compensated sensor and a simplified diagram of its calibration and field application. In Fig. 1A, a physical phenomenon emits a signal which travels to the sensor, in Fig. 1C, where it creates a measurable electric potential difference (voltage) across a transducer (Fig. 1D). The sensor circuits amplify and filter this voltage signal, and then feed it to an analog-to-digital converter (ADC) which digitizes it. This digital form is the raw output of the sensor and not the desired physical quantity (measurand): it needs an additional calibration-compensating mathematical transformation.

The sensor’s digital output and the measurand value follow a correspondence that depends on material- and manufacturing-related quantities. Estimating this correspondence is an important part of sensor calibration^11^. The outputs of the calibration process enable the construction of the inverse correspondence as a function for converting the raw digital sensor output to an estimate of the measurand. Such functions are commonly called *conversion routines* or *calibration-compensation routines*, and the calibration outputs are called *calibration parameters*.

Because of material and manufacturing variations, the exact calibration parameters between different instances of the same sensor are typically different. For this reason, sensor devices usually include a digital non-volatile memory that holds information about the calibration parameters—we refer to that information as *calibration data*. Sensor driver functions that recover calibration parameters from calibration data are called *extraction routines*.

Low-cost manufacturing and miniaturization impose strict size and power constraints on sensors. To conserve space and energy, the calibration data is a representation of the calibration parameters that uses less information. This work shows how this treatment of the calibration data affects the sensor output uncertainty in a significant measurable way.

Due to fundamental reasons of how calibration-compensated sensors work, there is unavoidable uncertainty about the sensor outputs, even if we assume a *perfect measurement* that has *zero noise*. This uncertainty is not due to ADC quantization (Fig. 1F) but because of deliberate data loss when storing coefficients in limited memory (Fig. 1K). Because of the dynamic nature of this uncertainty, sensor systems require a dynamic real-time approach to accurately quantify it.

This article provides a practical solution for on-device real-time quantification of dynamic uncertainty arising from pre-stored calibration data by making the following contributions to the state of the art:

① Demonstration of hardware and algorithmic techniques for the quantification of dynamic uncertainty in sensor outputs, with practical demonstration on two low-power FPGA-based hardware implementations of processor-native uncertainty tracking^12^. These present a way to improve the status quo, enabling more informed decision-making in critical applications where the reliability of sensor data is paramount.
② An analysis of epistemic uncertainty in the measurements of calibration-compensated sensors using a state-of-the-art infrared sensor (MLX90640) as a driving example. This epistemic uncertainty originates from the representation uncertainty of calibration numbers stored in-sensor and serves as the ground truth for the on-device approach.
③ Two experimental proof-of-usefulness examples: an edge detection application where making use of the uncertainty information leads to improved accuracy and precision, and an application in sensor design for faster design feedback cycles, which examines the output uncertainty for four different calibration data storage scenarios.
④ An extensive dataset of raw infrared measurements from four MLX90640 instances amounting to a total of 322,560 temperature readings of a high-accuracy, high-precision infrared calibration source, which we make public.

Our method and insights are applicable to sensors and devices that use conversion routines with calibration data, or other quantized pre-stored information. These insights are important for modern sensors, which use increasingly complex processes for measuring phenomena and involve more nonlinear elements^3,13,14^ while also pushing the boundaries of power consumption and portability^15^. Related work on sensor uncertainty characterization often lumps representation uncertainty together with other errors in a “systematic errors” category despite representation uncertainty being straightforward and worthwhile to quantify explicitly^11,16^, as this work shows.

### Representation uncertainty

Finite-precision representations map ranges of values in a dense domain to a single stored value, which discards information and introduces *representation error* (e.g., rounding or truncating a floating-point value to an integer). Because the mapping is many-to-one, the pre-conversion value becomes unknowable from the stored value alone. This work models the plausible pre-conversion values as a uniform distribution over the quantization bin, and calls that *representation uncertainty*. This uncertainty matters when devices quantize calibration coefficients for non-volatile storage and later reconstruct them at run time, since signal-path quantization-mitigation methods do not add information to stored coefficients, and reuse of uncertain values in later expressions can introduce correlation that complicates closed-form propagation. Supplementary Note 2 and Supplementary Note 3 further discuss discretization and representation uncertainty in calibration-compensated sensor systems.

## Discussion

This work introduces techniques for real-time uncertainty quantification in sensing systems and uses a calibration-compensated sensor as a driving example.

### Real-time on-device sensor uncertainty computation

The conventional approach for dealing with representation uncertainty involves the theoretical off-line study of the subject system to determine the uncertainty characteristics as constants which usually represent the worst case scenario across the operating domain (or a small amount of domain regions). Determined engineers apply compute-intensive Monte Carlo methods for fine-grained insights about the uncertainty characteristics of the system^17,18^. The Monte Carlo approach while effective, is too compute-intensive and too slow to deploy on actual real-time devices.

This article presents realistic on-device real-time uncertainty quantification in sensing systems which builds on recent advances in deterministic arithmetic with probability distributions (UxHw)^19^. We use two different hardware system-on-module implementations of an uncertainty-extended processor microarchitecture^12^ to benchmark the proposed UxHw approach. Instead of compute-intensive Monte Carlo simulations, the UxHw system uses deterministic arithmetic on fixed-size uncertainty representations to propagate uncertainty through computer operations, yielding deterministic runtimes and memory usage.

In contrast with bootstrap-based approaches which apply non-parametric bootstrap resampling to per-input ensembles of outputs produced by a conventional estimator to compute uncertainty statistics^17^, our proposed approach applies the UxHw system to propagate the parametric representation uncertainty of pre-stored calibration parameters through the calibration-extraction and calibration-compensation code, yielding an approximate uncertainty distribution for each calibration-compensated sensor output.

**Fig. 2:**
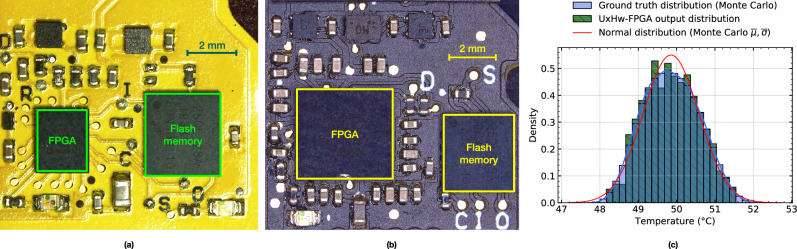
Uncertainty-tracking hardware modules and uncertainty quantification output. **a** Commercially-available system-on-module UxHw-FPGA-5k for native uncertainty tracking with 12 MHz clock speed, 128 KiB RAM, 12 mW base power, 10  × 15 mm size. **b** Commercially-available system-on-module UxHw-FPGA-17k for native uncertainty tracking with 45 MHz clock speed, 320 KiB RAM, 99 mW base power, 10  × 15 mm size. **c** Conversion routine output distribution when running on UxHw-FPGA-5k and UxHw-FPGA-17k with native uncertainty tracking. This result approximates the corresponding output distribution from the Monte Carlo execution with a Wasserstein distance of 0.0189 °C, in under 5 s per pixel and with average power 16.7 mW on UxHw-FPGA-5k. By contrast, to ensure same level of accuracy with 90% confidence using Monte Carlo execution, the embedded system must re-execute the conversion routines at least 3000 times, which takes 203 s.

Figure 2a, b show the two evaluation platforms, which this article refers to as UxHw-FPGA-5k and UxHw-FPGA-17k, respectively. UxHw-FPGA-5k builds on RISC-V RV32I and is optimized for low power but is also slower. UxHw-FPGA-17k, which builds on RISC-V RV32IM, is optimized for performance and is about ten times faster, but also consumes more power. On these two hardware platforms, we perform real-time dynamic uncertainty quantification for the calibration extraction and calibration-compensation code.

The following subsections take a closer look at the arithmetic involved in calibration-compensation of raw sensor outputs for a low-power infrared sensor which we use as a motivating application for our approach. For a target object at 50 °C, Fig. 2c presents the resulting output uncertainty of a single pixel, quantified by the Monte Carlo approach (blue histogram) and by our proposed UxHw approach (green histogram). The red line represents a normal distribution of the same mean and variance as the Monte Carlo data and highlights how this uncertainty follows a non-normal distribution.

### Conversion or calibration routines

Calibration-compensation routines are often provided by the sensor manufacturer in the form of computer code. This article uses the Melexis MLX90640 thermal sensor^5^ as a driving example to demonstrate real-time on-device uncertainty quantification of epistemic uncertainty which arises in calibration-compensated sensors because of the representation uncertainty in the calibration data.

As part of calibration compensation, the driver code must convert a raw 18-bit ADC reading of, e.g., 00 00000101 11011000 (1496) to an interpretable and useful quantity (in this case: 159.35 °C). To be able to convert the raw transducer reading to a temperature estimate for each pixel, the code first reads pre-stored calibration data from the non-volatile memory on the sensor device and applies the extraction routines to get the calibration parameters. For this conversion, the code uses 37 calibration parameters, four of which have different values for each of the 768 pixels of the sensor.

### Motivating example using infrared sensor

The MLX90640 has a thermal sensor array which senses radiation in the far-infrared range (FIR; 15 μm to 1 mm wavelength) with pixel resolution 32 × 24^5^. The pixel-sensors use thermopiles which transduce temperature differences to voltage via the Seebeck effect^20^.

Let *α* be the pixel sensitivity, *S* be the thermoelectric (Seebeck) effect coefficient slope^20^, and let *ϵ* be the target material emissivity. Let *c*_0_ = 273.15 K (0 °C). Let *V*_out_ represent the analog output voltage of a thermopile-based infrared sensor. Equation (1), adapted from the sensor datasheet^5^, gives the target object temperature (*T*_*o*_; in the Kelvin scale) for one pixel of the frame of the sensor.

1 $$\begin{array}{l}{T}_{o}=\root{{4}}\of{\frac{{V}_{out}}{\alpha S(\root{{4}}\of{\frac{{V}_{out}}{\alpha }+{T}_{a-r}}-{c}_{0})}+{T}_{a-r}},\\ {\mbox{where}}\enspace{T}_{a-r}=-\frac{(1-\epsilon ){T}_{r}^{4}}{\epsilon }-\frac{{T}_{a}^{4}}{\epsilon }.\end{array}$$

Supplementary Note 1 and Supplementary Discussion provide further context about the relevant physics as well as raw and extracted values and associated uncertainty for all calibration parameters (Supplementary Table 3 and Supplementary Table 4).

### Calibration data extraction

Each MLX90640 sensor has its own calibration data which the manufacturer stored on the sensor. Because the extraction routines have non-linear terms, uncertainty quantification becomes more difficult. As an illustrative example of a parameter extraction, let *α*_*i*_ be the calibration parameter related to the sensor sensitivity for pixel *i*. Let *A*_ref_, *R*, *C*, and *D* refer to calibration data which have representation uncertainty because they were quantized and stored in the device memory. Let *s*_*R*_, *s*_*C*_, and *s*_*D*_ refer to respective scales (non-uncertain calibration data). Let TGC represent the thermal gradient coefficient and *α*_CP_ compensation pixel values. TGC and *α*_CP_ are other calibration parameters with their own extraction routines and the extraction of *α* depends on their extracted values. Equation (2) is the mathematical equation for the extraction routine for *α*_*i*_, where *i* is the pixel index.

2 $${\alpha }_{i}=\frac{{A}_{ref}+{2}^{{s}_{R}}{R}_{row(i)}+{2}^{{s}_{C}}{C}_{col(i)}+{2}^{{s}_{D}}{D}_{i}}{{2}^{{s}_{\alpha }}}-TGC\cdot \frac{{\alpha }_{CP0}+{\alpha }_{CP1}}{2}.$$

### Quantifying representation uncertainty in MLX90640

The calibration data fundamentally represent real-valued quantities that the calibration process converted to integers to fit them in the sensor memory. Because of this, the calibration data are uncertain to the degree that their integer representation cannot fully represent the before-conversion numbers. Using floating-point representations for calibration data would partly mitigate but not eliminate the issue. We quantify this representation uncertainty as a uniform distribution with unit support length, centered on the raw calibration data.

**Fig. 3:**
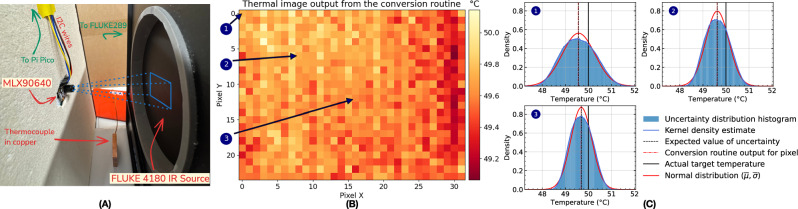
Quantification of representation uncertainty in the infrared sensor. **A** Experimental measurement setup: an MLX90640^5^ thermal sensor, controlled by a microcontroller, measures infrared radiation emanating from a FLUKE 4180 infrared calibration source. A FLUKE 289 thermocouple monitors ambient temperature. Red: key items. Green: supporting items, out of frame. Blue: sensor field-of-view projection. **B** The thermal image sensor outut of the setup in 3.A, after conversion. **C** Distribution of due-to-limited-precision uncertainty for three selected pixels of Fig. 3. Our method uncovers the dynamic uncertainty distribution of estimated temperature for every pixel of the image.

Figure 3A presents a physical measurement setup analogous to the diagram in Fig. 1. For one measurement using the setup, Fig. 3B shows the thermal image output of the manufacturer’s conversion routines. For three pixels along the main diagonal of the thermal image (top-left, halfway to the center, and center), Fig. 3C shows the probability distribution of the pixel temperature. This distribution is due to epistemic uncertainty that is a direct effect of the reduced information of the calibration data and we computed it by off-line off-device Monte Carlo simulation. Figure 3C visually shows how the distributions are non-uniform and different from Gaussians of the sample mean and variance.

**Fig. 4:**
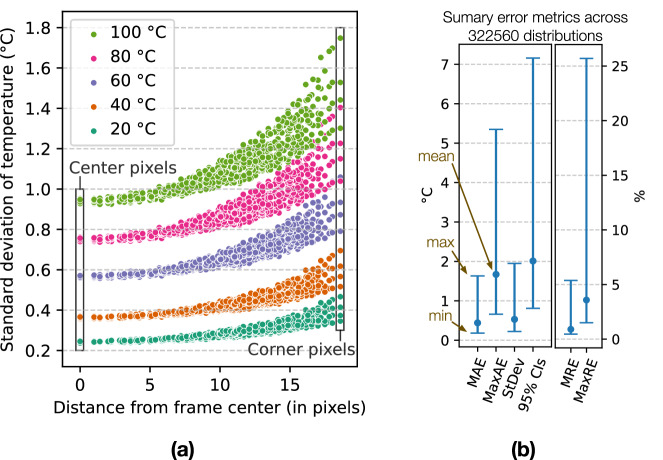
Representation uncertainty metrics for the infrared sensor. **a** The standard deviation of conversion routine output temperature for all 768 pixels of a single measurement of a single MLX90640 sensor instance. For a target temperature of 100 °C, the output uncertainty has average standard deviation 0.94 °C for the center pixels, while for the corner pixels std average 1.5 °C and max 1.75 °C. Overall, the standard deviation has an increasing trend as pixels approach the frame edge of the sensor output and as the temperature increases. **b** Summary for the probable absolute and relative errors because of representation uncertainty of the MLX90640 calibration data (conventional sensor output vs distribution), from all 768 pixels of four tested sensor instances, each tested for 21 target temperatures (322560 sensor output distributions). MAE: Mean Absolute Error, MaxAE: Max Absolute Error, StDev: Standard Deviation, 95% CIs: Size of 95% Confidence Intervals, MRE: Mean Relative Error, MaxRE: Max Relative Error. Error bars show the max and min values.

Figure 4a shows how this uncertainty is different for each pixel and temperature and Fig. 4b summarizes probable absolute and relative error metrics due to the sensor output uncertainty.

Because the extracted calibration parameters are functions of the raw calibration data, the parameters are also uncertain, and their uncertainty is a function of the raw calibration data uncertainty. To quantify this effect, we ran a 500K-iteration Monte Carlo execution of the conversion routines for each measurement and each pixel. Figure 4a shows how the standard deviation of uncertainty of the MLX90640 conversion routine output increases with the pixel-distance from the image center from 0.24 °C for center pixels at target temperature of 20 °C, to as high as 1.75 °C for corner pixels at target temperature of 100 °C. Figure 4b presents aggregate metrics for all 322560 the MLX90640 output pixel distributions. The probable maximum absolute error can be as high as 5.35 °C (or more), and as high as 25.7% max relative error (or more). The standard deviation can be as high as 1.9 °C and the 95% confidence interval can be as high as 7.16 °C. The representation uncertainty of the calibration parameters causes sensor output uncertainty to be greater than the nominal sensor accuracy of  ± 3 °C^5^.

Supplementary Note 2, Supplementary Note 3, and Supplementary Discussion further discuss representation uncertainty in the calibration data of the MLX90640.

### Applicability to other sensors and systems

This work examines the particular thermal imaging sensor as a driving example to show the effect of epistemic uncertainty in the sensor outputs and to present the hardware-based framework for its on-device dynamic computation. The presented framework extends to every sensor and system which uses calibration data stored on the device. Calibration data which is originally real numbers and is ultimately stored in a numerical domain of finite precision is unavoidably subject to representation uncertainty. This even applies to cases where the calibration-compensation is part of digital in-sensor processing before and not in the host microcontroller. In the context of the UxHw approach aiding device design-space exploration, the Results examine how different storage strategies affect this uncertainty which is, however, always there.

Modern sensors are increasingly more complex and include digital logic for calibration compensation which depends on the sensor device storing data from factory calibration. This same pattern shows up in environmental sensors^6–9,21^, inertial sensors^10,22–24^, and others^25–27^.

## Results

We quantify the accuracy of the approximate uncertainty quantification of the uncertainty-tracking hardware as the Wasserstein distance^28^ of the output distributions from the hardware to the Monte Carlo execution output. Intuitively, the Wasserstein distance measures the cost of transforming one distribution into another. Let two one-dimensional empirical distributions *P* and *Q* and let *F*_*P*_ and *F*_*Q*_ be the respective cumulative density functions. Equation (3) defines the first Wasserstein distance *W*_1_(*P*, *Q*) with the difference of *F*_*P*_ and *F*_*Q*_.

3 $${W}_{1}(P,Q)=\int \,| {F}_{P}(x)-{F}_{Q}(x)| dx.$$

The uncertainty information of both UxHw-FPGA-5k and UxHw-FPGA-17k achieves Wasserstein distance 0.0189 °C to the output distribution from the ground-truth 500K-iteration Monte Carlo execution. We compare the speed of the hardware uncertainty tracking of UxHw-FPGA-5k and UxHw-FPGA-17k against the speed of a brute-force Monte Carlo execution of equal accuracy on the same hardware but without uncertainty-tracking. Since an actual deployment cannot check the Wasserstein distance against some large ground truth dataset at runtime, it must make a Monte Carlo iteration count cutoff based on offline analysis of empirical data: we report the time it takes to run the Monte Carlo execution with iteration count cutoff which 90% of the time yields Wasserstein distance 0.0189 °C (i.e., worse distance 10% of the time). We refer to these as EqMCp90-FPGA-5k and EqMCp90-FPGA-17k. A higher percentile confidence for convergence requires even more compute time in the EqMCp90-FPGA Monte Carlo executions.

Variant UxHw-FPGA-5k with average power 16.7 mW runs in under 4740 ms per pixel while the equal-accuracy brute-force EqMCp90-FPGA-5k needs at least 203509 ms (42.9× speedup of UxHw over corresponding EqMCp90). Variant UxHw-FPGA-17k runs in 350 ms per pixel with average power under 147.15 mW, while the equal-accuracy brute-force computation needs at least 33042 ms (94.4× speedup of UxHw over corresponding EqMCp90). Table 1 lists the power dissipation of extracting the calibration parameters and their uncertainty, and computing the pixel output temperature and its uncertainty (excluding I2C sensor communication). Table 2 lists workload latency for the two hardware variants and their corresponding equal-accuracy Monte Carlo execution, and the speedup the UxHw-FPGA variants achieve over the corresponding EqMCp90-FPGA variant.

**Table 1:** Power dissipation measurements for FPGA-5k and FPGA-17k, the base platforms for UxHw-FPGA-5k and UxHw-FPGA-17k and the corresponding EqMCp90-FPGA-5k and EqMCp90-FPGA-17k executions

	Idle power	Average power	Max power
FPGA-5k	12 mW	17 mW	35 mW
FPGA-17k	99 mW	147 mW	208 mW

The low power dissipations verify our method as a valid approach for uncertainty quantification for sensor outputs on low-power edge devices. Bold typeface is headers.

**Table 2:** Iso-quality computation latency measurements for UxHw-FPGA-5k and UxHw-FPGA-17k and the corresponding EqMCp90-FPGA-5k and EqMCp90-FPGA-17k executions, for the same Wasserstein distance 0.0189 °C to the ground truth Monte Carlo execution output

	Latency (ms)	Speedup
**EqMCp90-FPGA-5k**	203,509	-
**UxHw-FPGA-5k**	4740	42.9×
**EqMCp90-FPGA-17k**	33,042	-
**UxHw-FPGA-17k**	350	94.4×

The uncertainty-tracking hardware variants achieve 42.9 × and 94.4 × speedup over the corresponding equal-accuracy brute-force Monte Carlo execution (on the same processor). Bold typeface is headers.

### Application: Edge detection on uncertain data

Detecting object edges in an image is a common process in imaging systems and is one of the steps necessary for object detection and identification^29,30^. As one demonstrative application of the impact of epistemic uncertainty in calibration-compensated sensors and the potential usefulness of tracking it in real time, we analyze how the repersentation uncertainty in the thermal data of the MLX90640 propagates through a common edge detection implementation: the Canny algorithm^31^. These effects of uncertainty all result from the manner of storage of the calibration data during the calibration process.

**Fig. 5:**
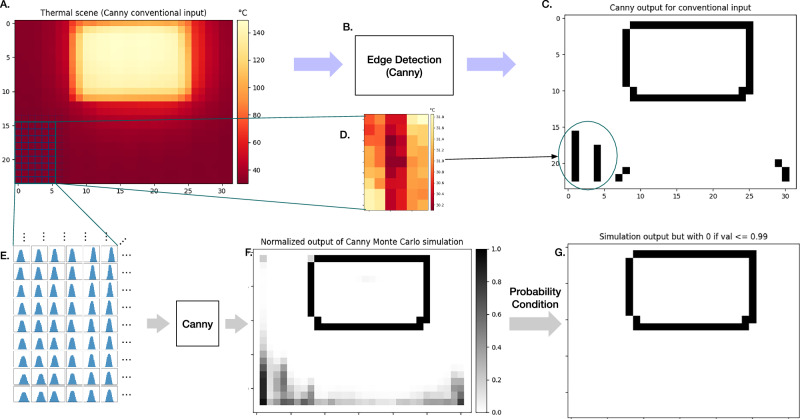
The uncertainty information helps against false positives in edge detection. **A** The conventional sensor output. **B** Canny edge detector. **C** The result of the Canny edge detector for the conventional sensor output yields false positive edges close to the bottom corners of the frame. **D** Small horizontal fluctuations leads to false positive edges. **E** The distribution of the temperature frame samples (see Discussion). **F** The result of the Canny execution using the temperature frame distribution yields probabilistic edges, with the highest probabilities for the true positive edges. False-positive edges in every sample frame: 26.8 ± 8. **G** The true edges have edge probability equal to one. We filter the data with probability lower than one and the true positive edges remain.

The top part of Fig. 5 shows how the application of the Canny detector on the sensor output for an edge detection scene (Fig. 5A) leads to false-positive edges in the output (Fig. 5C). The Canny algorithm uses a gradient-based operator and then decides which pixels are edges. The false-positive edges occur because of small gradients in the otherwise quiet part of the image (Fig. 5D).

We ran a 500K-iteration Monte Carlo execution of the Canny algorithm where each input sample is one sample-frame of 768 pixel temperatures—Fig. 5E shows this input to the Canny algorithm. Figure 5F shows the normalized aggregate result that represents the probability of Canny identifying each pixel as an edge, across the probable input temperature frames. Applying the conventional algorithm on different probable temperature frames leads to artifactual false-positive edges every time. The mean count of false-positives per sample frame is 26.8 pixels (i.e., 3.5% of the picture is falsely characterized as edges), with standard deviation 8.0, and max count 57 (i.e., 7.4% of the picture). Figure 5G shows how we can now filter out these false-positives by using a high-probability constraint, e.g., requiring more than 0.99 probability of edge to accept it.

We repeat this process for a total of ten scenes of varying complexity, emissivity, and background uniformity, and we use three empirical probability thresholds to quantify changes in the performance of the edge classification. The results show that the utilization of the epistemic uncertainty information as part of the Canny algorithm lead to improvements in accuracy and precision at the expense of sensitivity. For the 80%-probability threshold the approach yields on average +6 percentage points in accuracy, +46.3 percentage points in precision, and −16.6 percentage points in sensitivity. Supplementary Fig. 5 contains visuals for three of the ten scenes, and Supplementary Table 5 lists detailed performance metrics per scene.

### Application: calibration memory size scenario exploration

The epistemic uncertainties in the sensor outputs arise from engineering choices made in the sensor design and calibration phase. In particular, the representation uncertainty of the calibration data is a direct effect of the on-device flash memory size and the rounding algorithm. This section overviews how the UxHw approach applies to sensor design with the potential of faster design feedback cycles by enabling faster choice–effect approximation for design choices such as the on-board memory and the calibration process.

The calibration data that lead to the sensor output uncertainties of Fig. 4b take up 1.5 KiB of the 1.625 KiB EEPROM. This section examines the effect on this representation uncertainty when increasing the memory limit and when using different data formats to store calibration data. We study the effect of the four following hypothetical scenarios of using more memory for the MLX90640 calibration data. TwoMoreBits: Use two more bits for each piece of calibration data: this increases the necessary memory by about 48% to 2.225 KiB. TwiceBits: Use twice the amount of bits for each piece of calibration data: this increases the memory by about 108% to 3.125 KiB. FP16: Use the 16-bit IEEE-754 floating-point data format to store each calibration data: this increases the memory by about 228% to 4.925 KiB. FP32: Use the 32-bit IEEE-754 floating-point data format (single precision floating point) for each piece of calibration data: this increases the necessary on-device memory by about 548% to 9.725 KiB.

In scenarios TwoMoreBits and TwiceBits the data format stays the same but the quantization regions are smaller and thus the representation uncertainty is also narrower. Scenarios FP16 and FP32 use the a uniform width across all pieces of calibration data while the original format assigns non-uniform fidelities to different categories of calibration data. We compare these scenarios against the NearestInteger baseline scenario.

**Fig. 6:**
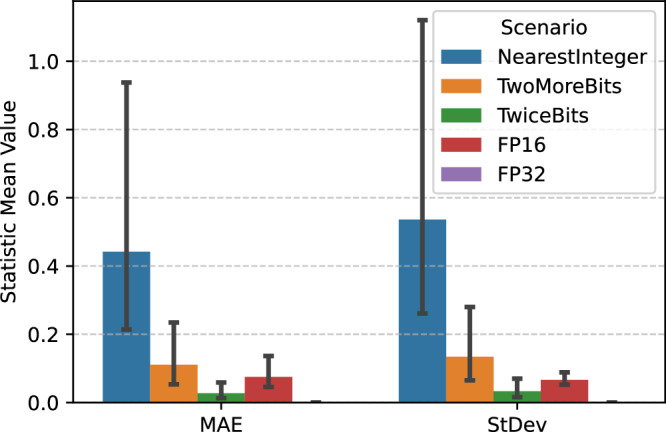
Mean absolute error and standard deviation statistic of the sensor output epistemic uncertainty distribution under the four examined calibration data storage scenarios. Error bars show the empirical 95% percentile interval.

Figure 6 shows the mean absolute error and standard deviation statistic of the sensor output epistemic uncertainty distribution under the four examined calibration data storage scenarios. Even using two more bits at the cost of 48% more memory requirements yields smaller possible due-to-quantization errors and narrower sensor output uncertainty distributions. Supplementary Table 6 presents finer-grained percent-change data for the summary statistics of the sensor output uncertainty. As expected, scenarios with more information storage capacity result to narrower epistemic uncertainty in the sensor outputs. The IEEE-754 32-bit scenario yields 99.99% smaller error statistics across all datasets. While the IEEE-754 16-bit requires more memory than the TwiceBits scenario, it yields less stable benefit compared to the nearest-integer rounding baseline.

These scenario simulations take hours to complete on a state-of-the-art machine. More sophisticated approaches would check for hybrid scenarios where calibration data is stored with non-uniform widths, as happens with the real calibration data on the MLX90640, and this means even more resource-intensive and time-consuming simulations. Engineers and designers can apply the UxHw method of this article for faster approximation of the effect of design choices on the output uncertainty and benefit from faster simulations and less time lost on design feedback cycles.

## Conclusions

In calibration-compensated sensor systems, real-valued calibration-related quantities undergo rounding as part of the calibration process that stores them as data in the sensor memory for later extraction. This process introduces representation uncertainty over the range of real numbers that rounding collapses to the same digital representation. This representation uncertainty leads to epistemic uncertainty in the sensor outputs. This uncertainty is an inherent fact of the physics and computation relevant to the sensor and does not come from noise in the measurement—it would be present even assuming perfect noiseless measurement. This epistemic uncertainty of the sensor output is not of a constant size or distribution shape. We cannot store it as an offset distribution and re-apply it for new measurements: For each pixel and raw temperature reading, the distribution is different. This uncertainty is also different based on the conversion technique of the calibration process.

The correct quantification of such uncertainty in conventional systems involves offline off-device analysis, often with compute-intensive Monte Carlo methods. This work employs processor-native uncertainty-tracking using UxHw to present a framework for on-device real-time dynamic uncertainty quantification. We show practical use of the framework with two commercial uncertainty-extended RISC-V processors and achieve uncertain quantification accuracy within 0.0189 °C Wasserstein distance to the Monte Carlo execution output, while also achieving 42.9× and 94.4× speedup compared to the equal-accuracy brute-force computation of using Monte Carlo execution. The hardware platforms achieve these results with as little as 35 mW and 208 mW, respectively, demonstrating the feasibility of the approach on small scale sensing systems.

We show two proof-of-concept usecases of the UxHw method for real-life applications to demonstrate how the uncertainty quantification framework of this work is practical and applicable for low-power embedded systems. The tracking of epistemic uncertainty in sensor outputs through downstream application computations enables better automated decision making: In the edge detection application, the uncertainty information helps greatly reduce false-positive edges. The dynamic quantification of this uncertainty can speed up design cycles and efficient design-space exploration by aiding trade-off decisions of embedded designs such as memory space.

### Future directions

Embedded systems with calibration-compensated sensors can rely on field calibration with higher-precision calibration data retention, when the sensing system can support it. Recent techniques from quantization-aware neural networks could help producing better-quality quantized fits directly^32^, while also using block floating-point or bfloat16 instead of unsigned integers^33^. Bayesian computing techniques may hold the key in uncertainty-aware sensor calibration^34^. Since sensor devices already bear unique identifiers, manufacturers could provide digital distribution channels for higher-precision calibration parameters.

## Methods

### Experimental measurement setup

Four MLX90640 (Adafruit breakboard) instances take five infrared measurements of a FLUKE 4180 high-precision calibration source from a distance of 10 cm at 21 temperature configurations. The calibration source emissivity is 0.95. We start at source temperature 10 °C and measure up to 110 °C with step 5 °C, with stabilization wait time 10 min. After stabilization, we place each sensor opposite across the source on an affixed vertical breadboard and take five infrared measurements. The breadboard ensures each sensor is in the same spot at the time of measurement. The field of view of the sensor is fully within the 15 cm diameter of the infrared source disk. Materials around the sensor position are shielded from infrared radiation using Superwool HT. A FLUKE 289 multimeter monitors the ambient temperature between the sensor and the source using a copper-stabilized thermocouple, outside the source’s field-of-view. (The MLX90640 also reports its own ambient temperature reading.)

A Raspberry Pi Pico WH 2022 with a RP2040 microcontroller^35^ running custom MicroPython code drives each MLX90640 sensor over I^2^C, first reading the EEPROM data and making five raw data measurements with sampling frequency 0.5 Hz (highest accuracy for MLX90640). The interpreter firmware is the official release RPI_PICO_W-20240602-v1.23.0.uf2. The Pico sends the data to a MacBook Pro M1 over Bluetooth LE. A Keithley 2281 precision DC supply powers the Pico at 5 V, which powers the MLX90640 from the 3V3 pin.

### Monte Carlo executions

Using the uniform uncertainty centered around each calibration data model, we do a Monte Carlo execution with 500000 re-executions of the conversion routines for all 768 pixels of four distinct MLX90640 sensor instances, and across five different actual measurements from each, each against 21 target temperatures settings of the infrared source. The Monte Carlo execution result data amount to 322560 empirical distributions total, each made up of 500000 samples—a total data size of 645.12 GB. We provide the input measurements as an open dataset and also open-source code that generates the result data in a way easily-configurable to simulate a subset of the results. Histogram plots use the Doane binning algorithm from NumPy^36^ unless specified otherwise.

### Benchmarking equipment

The Monte Carlo executions ran on a Dell Precision 5820 workstation with Ubuntu 24.04 LTS, Linux 6.11, Python 3.12, 16-core Intel i7-7820X 4.5 GHz, and 32 GiB RAM, as well as on a MacBook Pro with Apple M1 Pro, 32 GiB RAM, Sequoia 15, Apple clang 16.0. The executions for the Results ran on commercially available FPGA implementations of the Laplace microarchitecture^12^ (see paragraph on hardware in Methods).

### Metrics

Let *s* be an index over the sensor instances, *t* over the tested temperatures, *m* over the per-instance per-temperature measurement repetitions, and *i* over the sensor pixels. We compute the aggregate metrics between the conventional outputs of the sensors, *x*_*s*,*t*,*m*,*i*_, and the corresponding sensor output distribution, ${Z}_{s,t,m,i} \sim {\{{z}_{s,t,m,i,j}\}}_{j=1}^{500000}$, which is the result of the Monte Carlo simulation of the conversion routines with representation uncertainty in the calibration data. All samples of *Z*_*s*,*t*,*m*,*i*_ correspond to probable true temperatures which, due to calibration data uncertainty, all yield the same measured temperature *x*_*s*,*t*,*m*,*i*_. For this reason, the relative-error metrics use *Z*_*s*,*t*,*m*,*i*_ as the true-value reference and *x*_*s*,*t*,*m*,*i*_ as the measured value. Equations (4) through (9) give the metric formulas:

4 $$MAE(s,t,m,i)={\mathbb{E}}\langle | {x}_{s,t,m,i}-{Z}_{s,t,m,i}| \rangle ,$$

5 $$MRE(s,t,m,i)={\mathbb{E}}\left\langle \left| \frac{{Z}_{s,t,m,i}-{x}_{s,t,m,i}}{{Z}_{s,t,m,i}}\right| \right\rangle ,$$

6 $$MaxAE(s,t,m,i)=\max (| {x}_{s,t,m,i}-{Z}_{s,t,m,i}| ),$$

7 $$MaxRE(s,t,m,i)=\max \left(\bigg| \frac{{Z}_{s,t,m,i}-{x}_{s,t,m,i}}{{Z}_{s,t,m,i}}\bigg| \right),$$

8 $$StDev(s,t,m,i)=\sqrt{\frac{1}{N-1}{\sum }_{j=1}^{N=500000}{({z}_{s,t,m,i,j}-{\mathbb{E}}\langle {Z}_{s,t,m,i}\rangle )}^{2}},$$

9 $${SizeCI}_{95 \% }(s,t,m,i)={Q}_{0.975}({Z}_{s,t,m,i})-{Q}_{0.025}({Z}_{s,t,m,i}).$$

### Code changes to MLX90640 driver for simulation

We assume that the original calibration process performed by the manufacturer, to generate the manufacturer-supplied calibration data, rounds the calibration data using the C round() function which rounds half-way cases away from zero. To compute the uncertainty of the calibration uncertainty we model the representation uncertainty of the calibration data and then forward-propagate it through the extraction routines. To achieve that, we simulate uniform additive noise in the range [−0.5, 0.5), and center it around the integer value of each calibration data out of the sensor memory. The introduction of this additive noise must happen: ❶ After *logical* bit shifting operations that aim to place the data in the bits of correct significance; ❷ after arithmetic operations aiming to restore the negative values domain; ❸ before any operations including variables with values dependent on other calibration parameters; ❹ before other arithmetic operations of multiplication or division on the data. (In theory, additions and subtractions with constant scalars do not affect the representation uncertainty.)

While computing different calibration parameters, the parameter extraction code reads some calibration data more than once. To achieve correct modeling of variable dependencies in the simulation, our simulation code makes sure to sample the representation uncertainty of these calibration data once and reuse the value during the same execution.

The official C driver re-discretizes some extracted calibration parameters to conserve dynamic memory at the time of operation. Because we are studying the effect of representation uncertainty to the output temperature image, we change the simulation code to treat that data as floating-point numbers and sidestep that particular code. See Supplementary Note 3 for further discussion on this.

### Canny

The edge detection code uses the Canny operator implementation from Python package scikit-image v0.24^37^. We configure for unit standard deviation Gaussian filter, and nearest mode for filtering the array borders. Higher standard deviation for the Gaussian filter leads to fewer false-positive edges but also reduces the accuracy of true-positive edges.

### Real-time uncertainty quantification hardware

We use two commercial implementations of processor-native uncertainty-tracking microarchitecture^12^ as systems-on-module on FPGA: the UxHw-FPGA-5k and the UxHw-FPGA-17k. The devices track uncertainty in computation for floating-point variables and are based on RISC-V RV32I and RV32IM. We compile C code for the uncertainty-tracking hardware using the publicly-provided online access to the toolchain. We configure the uncertainty-tracking hardware for uncertainty-tracking computation with the smallest-available uncertainty representation (C0-microSD-N).

### Accuracy-based speedup benchmarking

To quantify the speedup of the UxHw approach we quantify how much faster it estimates the sensor output distribution. To do this, we first quantify the accuracy of the UxHw output in terms of the Wasserstein distance to the 500000 Monte Carlo execution result. Then, we perform batches of independent Monte Carlo simulations where each stops when it achieves the Wasserstein distance. The result is the empirical distribution of the Monte Carlo iteration counts necessary to beat UxHw. From this distribution, we pick the 90-th percentile that we call *EqMCp90* to time against UxHw on the FPGA-5k and 17k.

## Supplementary information

Transparent Peer Review file

Supplementary Information for Digital Methods to Quantify Sensor Output Uncertainty in Real Time

## Data Availability

The dataset of raw infrared measurements is available on Zenodo with 10.5281/zenodo.19387597.

## Code availability

The modified MLX90640 driver for the dynamic quantification of uncertainty in the sensor outputs is available on Zenodo with 10.5281/zenodo.19672271.

## References

[1] Chun, S. et al. An artificial neural tactile sensing system. *Nat. Electron.* **4**, 429–438 (2021).

[2] Yang, Y. et al. In-sensor dynamic computing for intelligent machine vision. *Nat. Electron.* **7**, 225–233 (2024).

[3] Lenk, C. et al. Neuromorphic acoustic sensing using an adaptive microelectromechanical cochlea with integrated feedback. *Nat. Electron.* **6**, 370–380 (2023).

[4] Shi, F. et al. Miniature optical fiber curvature sensor via integration with gan optoelectronics. *Commun. Eng.* **1**, 47 (2022).

[5] *Melexis MLX90640 32x24 IR array datasheet*. Melexis, Revision 12, 03/12/2019 (2019).

[6] *BME680: Low power gas, pressure, temperature & humidity sensor*. Bosch Sensortec, 2 Datasheet rev. 1.9 (2024a).

[7] *BME280 Combined humidity and pressure sensor*. Bosch Sensortec, 2 Datasheet rev. 1.24. (2024b).

[8] *MS5611-01BA03 Barometric Pressure Sensor, with stainless steel cap*. TE Connectivity, 1 (2017).

[9] *MPL115A2 Miniature I2C digital barometer*. NXP, 3 Datasheet rev. 10.1. (2024).

[10] *ADIS16470 Wide Dynamic Range Miniature MEMs IMU*. Analog Devices, Datasheet rev. C. (2019).

[11] Fraden, J. *Handbook of Modern Sensors: Physics, Designs, and Applications* 5th edn (Springer). 10.1007/978-3-319-19303-8 (2016).

[12] Tsoutsouras, V. et al. The Laplace microarchitecture for tracking data uncertainty and its implementation in a RISC-V processor. In *MICRO-54: 54th Annual IEEE/ACM International Symposium on Microarchitecture*, MICRO ’21, pages 1254–1269, New York, NY, USA, Association for Computing Machinery. 10.1145/3466752.3480131 (2021).

[13] Potyrailo, R. A. et al. Extraordinary performance of semiconducting metal oxide gas sensors using dielectric excitation. *Nat. Electron.* **3**, 280–289 (2020).

[14] Nakata, S. et al. A wearable pH sensor with high sensitivity based on a flexible charge-coupled device. *Nat. Electron.* **1**, 596–603 (2018).

[15] Maag, B., Zhou, Z. & Thiele, L. A survey on sensor calibration in air pollution monitoring deployments. *IEEE Internet Things J.* **5**, 4857–4870 (2018).

[16] Ru, X., Gu, N., Shang, H. & Zhang, H. MEMS inertial sensor calibration technology: current status and future trends. *Micromachines* **13**, 879 (2022). 35744491 10.3390/mi13060879 PMC9228165

[17] Zhou, K. et al. Real-time uncertainty quantification for KLT-based displacement estimation. *Eng. Struct.* **339**, 120671 (2025).

[18] Zhao, J. & Pan, B. Uncertainty quantification for 3D digital image correlation displacement measurements using Monte Carlo method. *Opt. Lasers Eng.* **170**, 107777 (2023).

[19] Bilgin, B. A., Elias, O. H., Selby, M. & Stanley-Marbell, P. Quantization of probability distributions viadivide-and-conquer: Convergence and error propagation under distributional arithmetic operations. *Methodol. Comput. Appl. Probab.*, **28**, 32 (2026).

[20] Van Herwaarden, A. W. & Sarro, P. M. Thermal sensors based on the seebeck effect. *Sens. Actuators* **10**, 321–346 (1986).

[21] *MS5837-30BA Ultra-small, gel-filled, pressure sensor with stainless steel cap*. TE Connectivity, 2 (2025).

[22] *AK8963 3-axis electronic compass*. AsahiKasei, 10 (2013).

[23] *ADIS16550 Autonomous Grade, Six Degrees of Freedom Inertial Sensor*. Analog Devices, Datasheet rev. 0. (2023).

[24] *LSM6DS33 iNEMO inertial module: always-on 3D accelerometer and 3D gyroscope*. ST, 10 (2015).

[25] *FLS110 Technical Note FL-000986-TN*. Flusso, 10 2021.

[26] *AS7263 6-Channel NIR Spectral_ID Device with Electronic Shutter and Smart Interface*. AMS, 11 (2016).

[27] *SFM3100 Data Sheet Low Pressure Drop Analog Flow Meter*. Sensirion, 1 (2021).

[28] Ramdas, A., García Trillos, N. & Cuturi, M. On Wasserstein two-sample testing and related families of nonparametric tests. *Entropy* **19**, 47 (2017).

[29] Shin, M. C., Goldgof, D. B. & Bowyer, K. W. Comparison of edge detector performance through use in an object recognition task. *Comput. Vis. Image Underst.* **84**, 160–178 (2001).

[30] Bansal, M., Kumar, M. & Kumar, M. 2D object recognition techniques: state-of-the-art work. *Arch. Comput. Methods Eng.* **28**, 1147–1161 (2021).

[31] Canny, J., A computational approach to edge detection. *IEEE Transactions on Pattern Analysis and Machine Intelligence*, 679–698 (1986). 21869365

[32] Jacob, B. et al. Quantization and training of neural networks for efficient integer-arithmetic-only inference. In *Proceedings of the IEEE Conference on Computer Vision and Pattern Recognition*, 2704–2713 (2018).

[33] Tye, N. J., Hofmann, S. & Stanley-Marbell, P. Materials and devices as solutions to computational problems in machine learning. *Nat. Electron.* **6**, 479–490 (2023).

[34] Berger, M., Schott, C. & Paul, O. Bayesian sensor calibration. *IEEE Sens. J.* **22**, 19384–19399 (2022).

[35] *RP2040 Datasheet*. Raspberry Pi Ltd, https://datasheets.raspberrypi.com/rp2040/rp2040-datasheet.pdf. Accessed: 2025-07-05, Version: 3184e62-clean. (2025).

[36] Harris, C. R. et al. Array programming with NumPy. *Nature* **585**, 357–362 (2020). 32939066 10.1038/s41586-020-2649-2 PMC7759461

[37] Pedregosa, F. et al. Scikit-learn: machine learning in Python. *J. Mach. Learn. Res.* **12**, 2825–2830 (2011).

