# Supplementary material for: Digital methods to quantify sensor output uncertainty in real time
Source: Commun Eng. 2026 May 13;5:135. doi: 10.1038/s44172-026-00679-4 (PMC13396527; doi:10.1038/s44172-026-00679-4)
Supplement: Supplementary file 2 — Supplementary Information for Digital Methods to Quantify Sensor Output Uncertainty in Real Time [file 44172_2026_679_MOESM2_ESM.pdf]

Supplementary Information for Digital Methods to Quantify Sensor Output Uncertainty in Real Time

Orestis Kaparounakis[a,†] **and Phillip Stanley-Marbell**[a,b,†]

[a]Physical Computation Laboratory, Department of Engineering, University of Cambridge, Cambridge CB3 0FA, UK.; [b]Signaloid, 4 Station Square, Cambridge, CB1 2GE, United Kingdom

[†]Correspondence may be addressed to any author. Orestis Kaparounakis: ok302@cam.ac.uk; Phillip Stanley-Marbell: ps751@cam.ac.uk.

Supplementary Information

Because contemporary digital computers are voltage-based, analog environmental information such as temperature or sound entering the computer system invariably pass through an electrical transducer to convert the original analog phenomenon to a voltage. And because the only way conventional digital computers convert voltage levels to digital values is to use an analog-to-digital converter (ADC), sensors in general transduce some physical phenomenon ultimately into a voltage.

The following sections provide further discussion and context around the core idea of representation uncertainty as well as Supplementary Discussion for the experimental validation using the Melexis MLX90640 far-infrared sensor array.

Supplementary Note 1: Infrared radiation and sensing. Infrared, or thermal, radiation is electromagnetic radiation in the infrared spectrum, i.e., wavelengths from 750 nm to 1 mm. It emanates from the surface of every material and has power proportional to the degree of thermal motion of the material's particles, colloquially known as the material's temperature.

The thermal emission power also depends on other material properties, e.g., its chemical composition[1]. An ideal object that emits the maximum possible thermal radiation at a given temperature is called a *black body*. Actual materials emit radiation at a fraction of the black-body radiation—that fraction is called *emissivity*. I.e., emissivity is a dimensionless quantity ranging from 0 to 1 and it quantifies a material's efficiency in emitting thermal radiation relative to an ideal black body.

Let T be the temperature of an object whose material has emissivity ϵ. Let P represent the total infrared radiation emission power per unit surface area, and let σ be the Stefan-Boltzmann constant. Supplementary Equation 1 states the Stefan-Boltzmann law which describes the relationship between the temperature of the object and the emission of infrared radiation.

$$P = \epsilon \sigma T^4. \tag{1}$$

Supplementary Equation 1 assumes constant emissivity across all wavelengths and temperatures. Some materials with low emissivity, such as commercial aluminum ($\epsilon \approx 0.04 - 0.09$), deviate from this assumption[2]. These materials predominantly reflect incident radiation rather than emit it, leading to mis-estimation of their true temperature when using infrared sensing techniques[3]. In such cases, the detected radiation may primarily originate from the surrounding environment rather than the object itself, rendering the simple Stefan-Boltzmann model inadequate. Accurate temperature measurement of low-emissivity materials necessitates more sophisticated models that account for factors such as environmental radiation reflection, wavelength-dependent emissivity, and surface conditions.

Thermopile-based infrared radiation sensors. Thermopile sensors capture electromagnetic energy in the infrared spectrum and convert it to electrical energy in the form of a measurable voltage via the thermoelectric (Seebeck) effect.

A thermopile consists of two or more thermocouples. A thermocouple is a temperature measurement device made of two dissimilar metals joined at two junctions. One junction receives the incoming radiation (hot junction) and the other is at a reference temperature (cold junction). This creates a temperature difference between the two junctions which results in a voltage potential due to the Seebeck effect.

Let S_{XY} be the Seebeck coefficient between metals X and Y. The temperature at the hot junction is T_H while at the cold junction is T_C. Supplementary Equation 2 shows the voltage output V of the thermocouple[4].

$$V = S_{XY}(T_H - T_C). \tag{2}$$

Individual thermocouple voltages are typically small, e.g., 58.7 μV K^{-1} for an ANSI Type E chromel-constantan thermocouple[5], one of the highest coefficients in the common junction types. Thermopiles use two or more thermocouple pairs placed in series, and these voltages add up. To increase sensitivity and utility, sensor package designs often include an amplification step before analog-to-digital conversion[6–9].

Let α be the pixel sensitivity and S be the Seebeck coefficient slope. Let $c_0 = 273.15$ K (0 °C). Let V_{out} represent the analog output voltage of a thermopile-based infrared sensor. Supplementary Equation 3, adapted from the sensor datasheet[10], gives the target object temperature (T_o; in the Kelvin scale) for one pixel of the frame of the sensor.

$$T_o = \sqrt[4]{\frac{V_{\text{out}}}{\alpha S(\sqrt[4]{\frac{V_{\text{out}}}{\alpha} + T_{a-r}} - c_0)} + T_{a-r}}. \tag{3}$$

Where the term T_{a-r} encodes the effect of ambient and reflected radiation on the sensor (Supplementary Equation 4).

$$T_{a-r} = -\frac{(1-\epsilon)T_r^4}{\epsilon} - \frac{T_a^4}{\epsilon}. \tag{4}$$

The manufacturer provides conversion routines to convert raw sensor readings (Figure 1.G) to per-pixel temperature values. These routines are functions of the 768-pixel raw sensor data, 37 calibration parameters (four of which are 768-element vectors), and two dynamic sensor-provided parameters (device temperature and voltage). Supplementary Table 3 lists the extracted value for all calibration parameters, and Supplementary Table 4 lists the raw calibration data.

Supplementary Note 2: Discretization introduces uncertainty. Real numbers are infinite but digital storage and memory are finite. Conversion of a number from a dense or continuous domain to a sparser domain maps regions of the originating domain, each to a single value in the target domain—the target domain has lower *precision.*

Representation error. Because of information loss, if after conversion we project a lower-precision quantity back to its original domain, the projection and the original quantity will almost certainly differ. Depending on the scientific field and the specific numerical domains involved, the difference between the original quantity and the projection is called quantization error, discretization error, or rounding error. This work refers to this error which arises due to conversion to a lower-precision representation as *representation error.* For example, in an application that requires large amounts of parameters (e.g., a machine learning algorithm), engineers may change the parameter representation size to reduce memory requirements, e.g., from 64 bit to 16 bit. This reduces the available information of the parameters and introduces representation error. This error is not because of noise in the original parameter values but rather an inherent principle of quantization.

Representation uncertainty. When converting quantities to a lower-precision domain, different numbers in the original domain end up as the same number in the target domain. Because conversion is a many-to-one function, we cannot know what the original number was before conversion. We therefore represent the probable original values as a uniform probability distribution around the converted value. This work refers to this probability distribution as *representation uncertainty.* For example, representation uncertainty arises in computer code when converting a floating-point value to an integer value via truncation or rounding.

Relation to quantization-mitigation methods. Engineers reduce quantization error in the *signal path*, e.g., at an ADC output or in repeated fixed-point arithmetic, by increasing bit depth, adding dither, oversampling, or using error feedback. Dithering, oversampling, and error-feedback corrections target quantization of time-varying quantities: they reduce deterministic artifacts and convert rounding effects into error terms that filtering suppresses. This article studies a different mechanism also present in sensors whereby devices quantize calibration data once to fit into limited non-volatile memory and then reuse those fixed bytes to reconstruct calibration parameters at run time. Applying these techniques does not increase the information content of stored coefficients. Repeated measurements do not reduce the resulting error unless the system performs online identification or stores side-information. Our analysis treats each stored datum as an unknown pre-quantization value within its quantization bin and dynamically propagates this representation uncertainty through the calibration-extraction and calibration-compensation code.

The Results section examines how different bit-depth choices for calibration data storage affect this uncertainty.

Representation uncertainty and correlation. Two or more uncertain quantities may share a relation—this means they share *mutual information* and are *correlated.* When inputs are correlated, uncertainty quantification must sample from the joint probability distribution of the quantities to account for the correlations [11].

Representation errors stemming from the discretization of different quantities are *mutually independent* [12]. However, correlations commonly arise during computation, even for independent inputs. This happens because an uncertain quantity may take part more than once in calculating the result—and at every operation it takes part in after the first, the operands will share mutual information. These runtime correlations make it hard to derive a closed-form solution for the uncertainty of computation, and analyses today often have to rely on computationally-expensive Monte Carlo methods.

Supplementary Note 3: Quantization and representation uncertainty examples. For a simple analog-to-digital conversion example, consider an 10-bit ADC, with 1024 levels of quantization. We use it to quantize a voltage input that can range from 0 V to 1 V. Supplementary Equation 5 is the input-output relationship of typical mid-tread quantization.

$$Q_{\mathrm{MT}}(x) = \Delta \cdot \left\lfloor \frac{x}{\Delta} + \frac{1}{2} \right\rfloor . \tag{5}$$

We adjust the ADC for $\Delta = 0.0009765625$, dividing the voltage range in 1024 regions. For $x = 0\,\mathrm{V}$, the ADC gives output 0, and for $x = 0.5\,\mathrm{V}$, 512. All voltages in the same region of size Δ result to the same integer. The digital system that receives the ADC output of 512 should heed that the ADC input could be any voltage in the region $[0.499\,511\,718\,75\,\mathrm{V}, 0.500\,488\,281\,25\,\mathrm{V})$—an epistemic uncertainty. If a function describes the relation of a physical quantity to the input voltage, the inverse function describes the propagation of the uncertainty about the input voltage to the uncertainty of the physical quantity. Because sensors transduce physical quantities to voltages, and so need a way to relate the raw output back to the physical quantity, this property is significant in to them.

Representation uncertainty as an effect of quantization via a type conversion arises in computer code that stores fundamental real-values quantities, eventually as integers in a storage device. For example, this happens during sensor calibration processes that store calibration parameters on-device as integers, as is the case with the MLX90640 sensor [10]. Because the difference between sequential integers is one ($\Delta = 1$ in Supplementary Equation 5), Supplementary Equation 6 is a representation of nearest-integer rounding that rounds halfway cases away from zero, as does the C function `round()`.

$$Q_{\mathrm{NI}}(x) = \left\lfloor x + \frac{1}{2} \right\rfloor . \tag{6}$$

One common method of sensor calibration is fitting an expected function type to the raw sensor data and ground truth data from a controlled calibration environment [13,14]. As a simple example, consider a linear fit with two coefficients, $ax + b$. Let $a^\star$, $b^\star$ be the ideal coefficients with minimum error between ground truth and prediction. Let a_{fit}, b_{fit} be the coefficient results of the fitting algorithm. Let a_{int}, b_{int} be the integer coefficients stored on the sensor device. For a physical model with $a^\star = 0.6$ and $b^\star = 3.4$, a fit on 100 data points with zero-mean Gaussian noise (with standard deviation 0.1) finds $a_{\mathrm{fit}} = 0.55782446$, $b_{\mathrm{fit}} = 3.3512181$, and with nearest-integer rounding $a_{\mathrm{int}} = 1$, $b_{\mathrm{int}} = 3$. This implementation uses `numpy.polyfit`, Python 3.12.8, and NumPy 2.1.1. Supplementary Figure 1 visualizes the example and the inaccuracy of the integer coefficients. In a more realistic scenario, calibration processes scale and offset the data to reduce the memory footprint of the calibration parameter and also achieve a more precise integer fit for two-parameter linear-fit scenario. However, calibration processes perform this space optimization for more than two parameters and for non-linear transformations, and inevitably cannot fully avoid this error.

Sensors that leverage coefficient-based fitting as part of the calibration process need to store the calibration data in a non-volatile memory of the sensor package because these data are different for

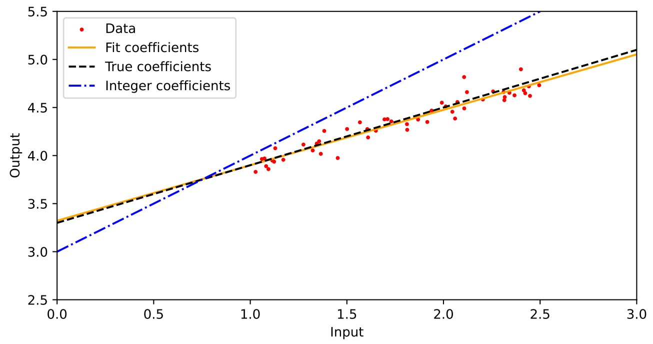

Supplementary Figure 1. Simplified example of sensor calibration using coefficients of a linear fit. While linear regression (yellow) may be sufficiently close to the true model (black), using the coefficients after rounding is an inaccurate model (blue). Sensors uses coefficient-based calibration need to store device-specific numbers in the constrained bit lengths of non-volatile memory.

each instance of the sensor. The storage encoding must be able to sufficiently represent the effective coefficient space for this particular sensor type and sensor manufacturers may provide coefficient extraction procedures to recover the coefficients (these procedures need to be the exact same for all instances of the sensor). Non-linear models with increased numbers of coefficients exacerbate this issue as they are inherently more sensitive to smaller changes in the coefficient values.

Rounding and truncation in C. The calibration process itself is computer code, often a program written in the C programming language. Before storing the calibration data in the memory of each device, the calibration program performs arithmetic operations and ultimately, for quantities that do not correspond to ordinal values, either casts the value to an integer, often as an implicit conversion (implicit cast), or explicitly rounds the value to the nearest integer. In the C language, when casting a floating-point number to an integer the implementation discards fractional part (truncation towards zero; Supplementary Equation 7)[15].

$$Q_{\mathrm{TTZ}}(x) = \mathrm{sgn}(\mathrm{x}) \lfloor |x| \rfloor . \quad (7)$$

Sensor calibration processes and software typically explicitly defines the rounding approach for the calibration parameters, which most often is nearest-integer rounding (Supplementary Equation 6). For some sensors, application engineers are able to perform their own calibration and store application-specific calibration data back to the sensor memory. This is the case for Bosch BNO055 where the official sensor driver includes functions for passing calibration data back to the sensor package as integers of type `signed short`. Those calibration data come from calibration parameters which are in the real domain. So, there is representation uncertainty when storing them as `signed short` (typically 16 bits in low-power systems). Also, the manufacturer does not clarify a rounding scheme for passing these parameters, and the different ranges of representation uncertainty associated with different rounding schemes leads to different sensor output uncertainty.

Tracking uncertainty across re-discretizations. The official C driver re-discretizes some extracted calibration parameters to conserve runtime memory usage. For example, it stores the extracted per-pixel α, $\mathrm{K_{Ta}}$, and $\mathrm{K_V}$ coefficients as integer numbers, after explicitly rounding them from floating point to the nearest integers. At the time of temperature conversion, the driver code performs a second set of ad hoc extractions for these parameters and then uses them in floating-point form. Because we are studying the effect of representation uncertainty to the output temperature image, we change the execution code to treat this data as floating-point numbers throughout execution, and sidestep the re-discretization.

This memory-conservation technique introduces a second level of representation uncertainty affecting the output of the sensor that warrants its own separate investigation. Probationary Monte Carlo executions for quantifying the effect of the memory-conservation technique showed that the change was much smaller compared to the base representation uncertainty of the calibration data. For the sake of brevity and clarity, and without real loss of generality, we bypass the specific part of the driver and do not quantify that effect in this work.

Supplementary Discussion. In case of sensors where use of calibration data is necessary, sensor manufacturers provide conversion routines to convert raw sensor readings to meaningful sensor outputs. For the MLX90640, these routines convert raw data to per-pixel temperature values, as exemplified by Figure 1(G) and Figure 1(L), respectively. These conversion routines are functions of the 768-pixel raw sensor data, 37 calibration parameters (four of which are 768-element arrays), and two dynamic sensor-provided parameters (device temperature and voltage). Supplementary Table 1 lists aggregate metrics for all 322 560 the MLX90640 output pixel distributions (recall Figure 4).

Supplementary Table 3 lists the extracted value for each calibration parameter. The official MLX90640 driver[16] extracts the raw calibration data from the sensor by reading the EEPROM memory. The driver firsta reads the data from the memory as unsigned 16-bit integer numbers (`uint16_t`). Then, it performs logical and arithmetic operations on the values read, such as bit shifts and floating-point scaling, to compute the calibration parameters—in some sense, the calibration parameters have their own set of conversion routines.

Supplementary Table 4 lists the raw scalar calibration data that is available in the sensor memory. Applications that need to extract the calibration parameters from this data are subject to the uncertainty of the quantized representation of the data. This is an effect of the calibration process that converts analog quantities or digital floating-point values ultimately to an integer to store in the sensor device memory. Using floating-point representations for calibration data would partly mitigate but not eliminate the issue.

The Uncertainty column of Supplementary Table 2 shows the rough outline of the representation uncertainty for five scalar calibration parameters relevant to the sensitivity calibration of the sensor. Because these uncertainties show up through operations that sometimes reference the value of other calibration parameters more than once, the uncertainty of calibration parameters can share mutual information. This happens even though these uncertainties originate from uncorrelated representation uncertainty of calibration data due to integer conversion[12]. For example, in Equation 2 the uncertainties of α_3 and α_4 share mutual information in A_{ref}, R_0, TGC, and α_{CP}. Supplementary Figure 2 further illustrates the extraction of the calibration parameter tensor α.

Supplementary Figure 3 shows the conventional values of the four extracted per-pixel calibration parameters. Each of these is a 768-element vector that for better intuition we plot as an image of the same dimensions as the sensor output. Supplementary Figure 4 presents the raw calibration data (from the sensor memory) which is the input for the extraction routines that compute the calibration parameters of Supplementary Figure 3.

Supplementary Table 1. Metrics summary for the output uncertainty of the MLX90640 from all 768 pixels of four tested sensor instances, each tested for 21 target temperatures (322 560 sensor output distributions). This is the same data as Figure 4b, in literal numerical format.

Metric aggregates	Min	Mean	Max
**Mean Absolute Errors (°C)**	0.18	0.44	1.63
**Mean Relative Errors**	0.46%	0.90%	5.38%
**Max Absolute Errors (°C)**	0.66	1.67	5.35
**Max Relative Errors**	1.50%	3.58%	25.70%
**Standard deviations (°C)**	0.22	0.53	1.95
**Size of 95% CIs (°C)**	0.81	2.01	7.16

CIs=Confidence Intervals

Uncertainty information and edge detection. As one demonstrative application of the impact of epistemic uncertainty in calibration-compensated sensing, and the potential usefulness of tracking it in real time, we examine the propagation of the representation uncertainty from the MLX90640 conversion routines through the Canny edge detection algorithm. We study ten different scenes of varying complexity, emissivity, and background. For each scene, we run a Monte Carlo simulations with 500K re-executions of the Canny algorithm. For each re-execution, the input sample is one probable sample-frame of 768 pixel temperatures from the MLX90640 sensor. The output of each simulation is a distribution of edge-annotated frames.

The rows of Supplementary Figure 5 show the thermal image output, the edge detection output using the conventional thermal image, and the edge detection output using the uncertainty-aware framework of this work. Supplementary Figure 5 shows these for three testcase scenes (columns): Calibration Source 60 °C which corresponds to an image of the infrared calibration source where all pixels are nominally the same temperature, a Mac Mini M4 that has heated up under load, and a USB hub which has heated up under heavy-traffic.

Supplementary Table 5 lists the performance metrics of the conventional and of the uncertainty-aware approaches

Uncertainty information and design-space exploration. Supplementary Table 6 presents detailed data for the percent-change differences of the sensor output uncertainty for each of the four alternative calibration-data storage scenarios.

Supplementary References

1. G Neuer and G Jaroma-Weiland. Spectral and total emissivity of high-temperature materials. *International Journal of Thermophysics*, 19(3):917–929, 1998.
2. *Melexis MLX90616ESF-HCA Infra Red Thermometer in TO-39 for high temperature thermometer guns.* Melexis, 2012. Revision 002.
3. Chang-Da Wen and Issam Mudawar. Emissivity characteristics of roughened aluminum alloy surfaces and assessment of multispectral radiation thermometry (MRT) emissivity models. *International Journal of Heat and Mass Transfer*, 47(17-18):3591–3605, 2004.
4. AW Van Herwaarden and PM Sarro. Thermal sensors based on the seebeck effect. *Sensors and Actuators*, 10(3-4):321–346, 1986.
5. *Standard Specification for Temperature-Electromotive Force (emf) Tables for Standardized Thermocouples.* American National Standards Institute (ANSI), astm e230/e230m-23a edition, January 2024.
6. *Thermopile Temperature Sensor (Model: MRT313) User Manual.* Zhengzhou Winsen Electronics Technology Co., Ltd, 2022.
7. Marek Lis. *How to Achieve Microvoltage-level Precision in Thermopile Applications.* Texas Instruments, 2021. URL https://www.ti.com/lit/ta/sszt108/sszt108.pdf.
8. *Thermopile detectors.* Hamamatsu, 2024. URL https://www.hamamatsu.com/content/dam/hamamatsu-photonics/sites/documents/99_SALES_LIBRARY/ssd/thermopile_kird9005e.pdf.
9. *NDIR Thermopile-Based Gas Sensing Circuit.* Analog Devices, 2016.
10. *Melexis MLX90640 32x24 IR array datasheet.* Melexis, 2019. Revision 12, 03/12/2019.
11. Christos E Papadopoulos and Hoi Yeung. Uncertainty estimation and Monte Carlo simulation method. *Flow Measurement and Instrumentation*, 12(4):291–298, 2001.
12. Manfred Drosg. *Dealing with uncertainties: A Guide to Error Analysis.* Springer, 2009. Section 3.6.
13. Daniel Trevisan Tatsch, Alejandro Rafael Garcia Ramirez, Fernando Campo, Leonardo Hoinaski, and Evelio González-Dalmau. An open-source tool for evaluating calibration techniques used in low-cost air pollutant monitors. *Electronics Letters*, 59(10):e12816, 2023.
14. Jacob Fraden. *Handbook of Modern Sensors: Physics, Designs, and Applications.* Springer, 5th edition, 2016. . URL https://link.springer.com/10.1007/978-3-319-19303-8.
15. *The C99 Standard ISO/IEC 9899:1999.* ISO/IEC JTC1/SC22/WG14, 1999.
16. Melexis. Mlx90640 library functions. GitHub Repository, 2019. URL https://github.com/melexis/mlx90640-library.

Supplementary Table 2. Representative subset of extracted calibration parameters for the Melexis MLX90640 infrared sensor conversion routines. The Uncertainty column documents which parameters we consider for epistemic uncertainty due to representation uncertainty in this work and its rough shape. The conventionally-extracted value appears in the uncertainty plots as a vertical red dashed line. Scale-related parameters such as s_α are essentially ordinal numbers stored without loss of precision. Supplementary Table 3 is the full table. Bold typeface is headers.

**Row**	**Calibration parameter**	**Extracted value**	**Uncertainty**	**Uncertainty type**
R8	TGC	0		Epistemic
R23	s_α	11	No (ordinal)	N/A
R26	α_{CP0}	$3.346\,940\,502\,524\,376 \times 10^{-9}$		Epistemic
R27	α_{CP1}	$3.425\,384\,420\,552\,291 \times 10^{-9}$		Epistemic
R35	α	Suppl. Fig. 2A	Pixel (12,16):	Epistemic

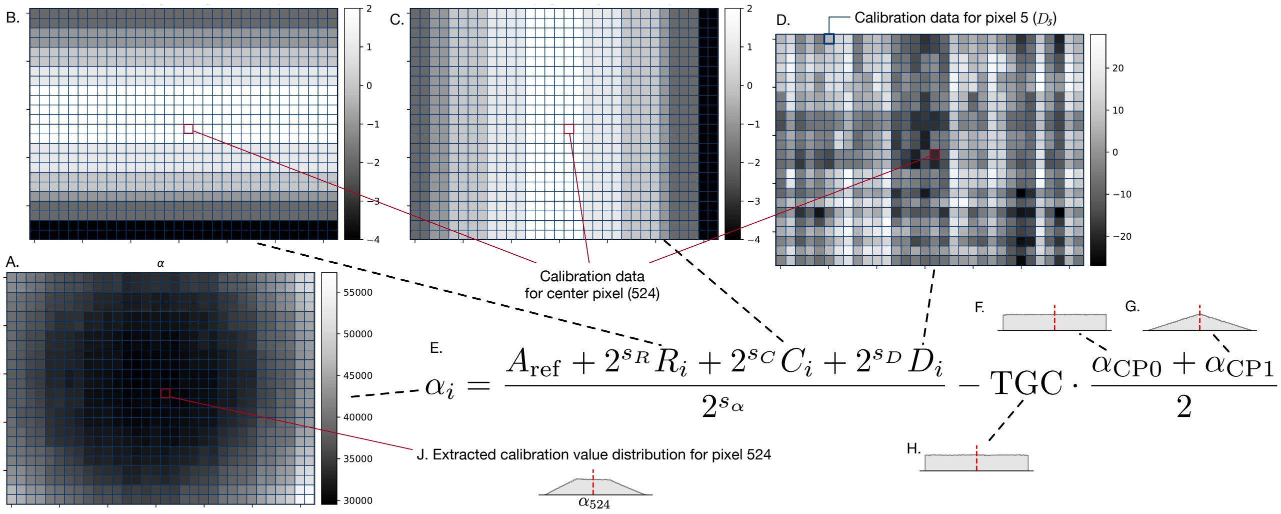

Supplementary Figure 2. Extraction of the sensitivity calibration parameter α (Supplementary Table 2.R35). A, The values of the extracted sensitivity calibration parameter α. It has different value for each pixel because the calibration data that takes part in the extraction equation has different values for each pixel. **B,** Per-row calibration data for α. **C,** Per-column calibration data for α. **D,** Per-pixel calibration data remainder for α. **E,** The mathematical extraction equation for calibration parameter α. **F**, **G**, and **H**, Representation uncertainty of already-extracted calibration parameters α_{CP0}, α_{CP1}, and TGC. **J,** Representation uncertainty of just-extracted sensitivity calibration parameter for the center pixel (a_{524}).

Supplementary Table 3. The calibration parameters for the Melexis MLX90640 infrared sensor conversion routines, after extraction from the device memory and applying the extraction routines. Column *Row* provides a reference numbering. Column *Calibration parameter* gives the name of each parameter. The conventional extracted value appears in the third column, as well as the rough uncertainty plots of column *Uncertainty* as a vertical red dashed line. Column *Uncertainty* documents which parameters we consider for epistemic uncertainty due to representation uncertainty in this work and its rough shape. Parameters without uncertainty are either categorical numbers stored without loss of precision or values the manufacturer has directly hardcoded in the sensor driver source code (i.e., they do not actually come from the sensor device memory). The "hardcoded" values have uncertainty that we are unable to confidently quantify with our method because the necessary information is not available. The last column gives the uncertainty type of the calibration parameter, where applicable. Bold typeface is headers.

**Row**	**Calibration parameter**	**Extracted value**	**Uncertainty**	**Uncertainty type**
R1	$K_{V_{DD}}$	−3040	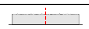	Epistemic
R2	V_{DD25}	−12 864	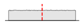	Epistemic
R3	$K_{V_{PTAT}}$	0.001 953 125	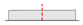	Epistemic
R4	$K_{T_{PTAT}}$	42.75	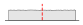	Epistemic
R5	V_{PTAT25}	12 194	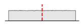	Epistemic
R6	α_{PTAT}	9	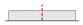	Epistemic
R7	GAIN	6276	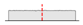	Epistemic
R8	TGC	0	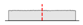	Epistemic
R9	$K_{V_{CP}}$	0.375	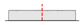	Epistemic
R10	$K_{Ta_{CP}}$	0.004 394 531 25	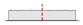	Epistemic
R11	Resolution_{EE}	2	No (nominal)	N/A
R12	$\text{CalibrationMode}_{EE}$	128	No (nominal)	N/A
R13	Ks_{Ta}	−0.001 220 703 125	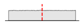	Epistemic
R14	$Ks_{To}[0]$	−0.000 396 728 515 625	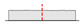	Epistemic
R15	$Ks_{To}[1]$	−0.000 450 134 277 343 75	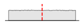	Epistemic
R16	$Ks_{To}[2]$	−0.000 602 722 167 968 75	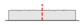	Epistemic
R17	$Ks_{To}[3]$	−0.000 801 086 425 781 25	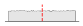	Epistemic
R18	$Ks_{To}[4]$	0.0	Not quantified (hardcoded)	Epistemic
R19	CT[0]	−40	Not quantified (hardcoded)	Epistemic
R20	CT[1]	0	Not quantified (hardcoded)	Epistemic
R21	CT[2]	120	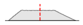	Epistemic
R22	CT[3]	240	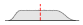	Epistemic
R23	s_{α}	11	No (ordinal)	N/A
R24	$s_{K_{Ta}}$	13	No (ordinal)	N/A
R25	s_{K_V}	7	No (ordinal)	N/A
R26	α_{CP0}	$3.667\,082\,637\,548\,446\,7 \times 10^{-9}$	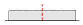	Epistemic
R27	α_{CP1}	$3.523\,837\,222\,019\,210\,5 \times 10^{-9}$	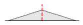	Epistemic
R28	$CP_{Offset}[0]$	−80	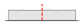	Epistemic
R29	$CP_{Offset}[1]$	−75	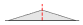	Epistemic
R30	$IL_{Chess_C}[0]$	0.9375	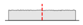	Epistemic
R31	$IL_{Chess_C}[1]$	4.0	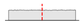	Epistemic
R32	$IL_{Chess_C}[2]$	0.0	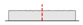	Epistemic
R33	BrokenPixels[]	Empty	No (ordinal)	N/A
R34	OutlierPixels[]	Empty	No (ordinal)	N/A
R35	α	Suppl. Fig. 2.A	Pixel (12,16): 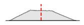	Epistemic
R36	Offset	Suppl. Fig. 3b	Pixel (12,16): 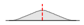	Epistemic
R37	K_{Ta}	Suppl. Fig. 3c	Pixel (12,16): 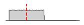	Epistemic
R38	K_V	Suppl. Fig. 3d	Pixel (12,16): 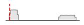	Epistemic

The values in the table above are from sensor instance with identifier `0x1f15cb2d0189`.

Supplementary Table 4. Calibration data from the flash memory of an Melexis MLX90640 infrared sensor device. Column *Row* provides a reference numbering. Column *Calibration data name* gives the name of the calibration data parameter. The same information appears in the MLX90640 datasheet as the "calibration parameters memory" table [10]. The symbol "±" means the number is in two's complement format. Column *Flash value* gives the calibration data value read from the sensor memory and column *Bit count* gives the data size in that memory. Column *Source register* documents the origin address of the data. The last column documents which calibration data we consider for representation uncertainty: we do not consider ordinal values (scaling factors), nominal values (interleaving pattern mode), or hardcoded values as having representation uncertainty. Bold typeface is headers.

**Row**	**Calibration data name**	**Flash value**	**Bit count**	**Source register**	**Considered uncertain**
R1	(Alpha PTAT - 8)*4	4	4	EE[0x2410]	Yes
R2	Scale OCC Row	2	4	EE[0x2410]	No
R3	Scale OCC Col	2	4	EE[0x2410]	No
R4	Scale OCC Rem	0	4	EE[0x2410]	No
R5	± Pix Os Average	65 466	16	EE[0x2411]	Yes
R6	Alpha Scale - 30	8	4	EE[0x2420]	No
R7	Scale ACC Row	9	4	EE[0x2420]	No
R8	Scale ACC Col	10	4	EE[0x2420]	No
R9	Scale ACC Rem	5	4	EE[0x2420]	No
R10	Pix Sensitivity Average	14 403	16	EE[0x2421]	Yes
R11	± Gain	6276	16	EE[0x2430]	Yes
R12	± PTAT 25	12 194	16	EE[0x2431]	Yes
R13	± Kv PTAT	8	6	EE[0x2432]	Yes
R14	± Kt PTAT	342	10	EE[0x2432]	Yes
R15	± Kv Vdd	161	8	EE[0x2433]	Yes
R16	± Vdd 25	110	8	EE[0x2433]	Yes
R17	± Kv Avg Row-Odd Column-Odd	4	4	EE[0x2434]	Yes
R18	± Kv Avg Row-Even Column-Odd	3	4	EE[0x2434]	Yes
R19	± Kv Avg Row-Odd Column-Even	3	4	EE[0x2434]	Yes
R20	± Kv Avg Row-Even Column-Even	3	4	EE[0x2434]	Yes
R21	± IL Chess C3	0	5	EE[0x2435]	Yes
R22	± IL Chess C2	8	5	EE[0x2435]	Yes
R23	± IL Chess C1	15	6	EE[0x2435]	Yes
R24	± Kt Avg Row-Odd Column-Odd	68	8	EE[0x2436]	Yes
R25	± Kt Avg Row-Odd Column-Even	60	8	EE[0x2436]	Yes
R26	± Kt Avg Row-Even Column-Odd	64	8	EE[0x2437]	Yes
R27	± Kt Avg Row-Even Column-Even	58	8	EE[0x2437]	Yes
R28	Res Control Calib	2	2	EE[0x2438]	No
R29	Kv Scale	3	4	EE[0x2438]	No
R30	Kta Scale 1	5	4	EE[0x2438]	No
R31	Kta Scale 2	2	4	EE[0x2438]	No
R32	± Alpha ((CP Subpage 1) / (CP Subpage 0) - 1)*2^7	59	6	EE[0x2439]	Yes
R33	Alpha CP Subpage 0	126	10	EE[0x2439]	Yes
R34	± Offset (CP Subpage 1 - CP Subpage 0)	5	4	EE[0x243A]	Yes
R35	± Offset CP Subpage 0	944	4	EE[0x243A]	Yes
R36	± Kv CP	3	8	EE[0x243B]	Yes
R37	± Kta CP	36	8	EE[0x243B]	Yes
R38	± KsTa*2^{13}	246	8	EE[0x243C]	Yes
R39	TGC (±4)*2^7	0	8	EE[0x243C]	Yes
R40	± KsTo Range 2 (0°C…CT1°C)	197	8	EE[0x243D]	Yes
R41	± KsTo Range 1 (<0°C)	204	8	EE[0x243D]	Yes
R42	± KsTo Range 4 (CT2°C…)	151	8	EE[0x243E]	Yes
R43	± KsTo Range 3 (CT1°C…CT2°C)	177	8	EE[0x243E]	Yes
R44	Temp Step x10	2	2	EE[0x243F]	Yes
R45	CT4	6	4	EE[0x243F]	Yes
R46	CT3	6	4	EE[0x243F]	Yes
R47	KsTo Scale Offset - 8	9	4	EE[0x243F]	No

The values in the table above are from sensor instance with identifier 0x1f15cb2d0189.

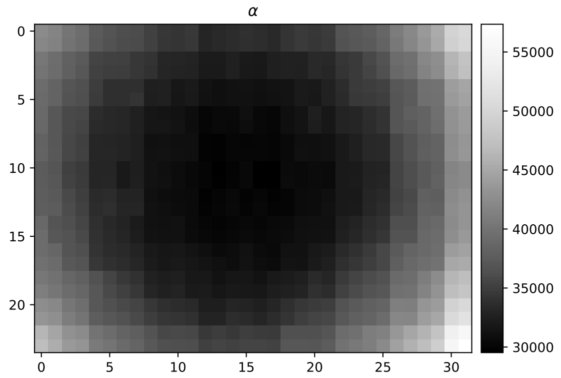

(a) The sensitivity calibration parameter α has different value for each pixel. Because of the representation uncertainty in the calibration data each α-pixel has a different probability distribution.

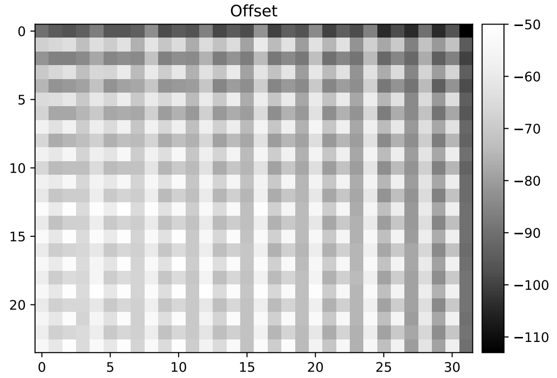

(b) The offset calibration parameter has different value for each pixel. Because of the representation uncertainty in the calibration data each pixel offset has a different probability distribution.

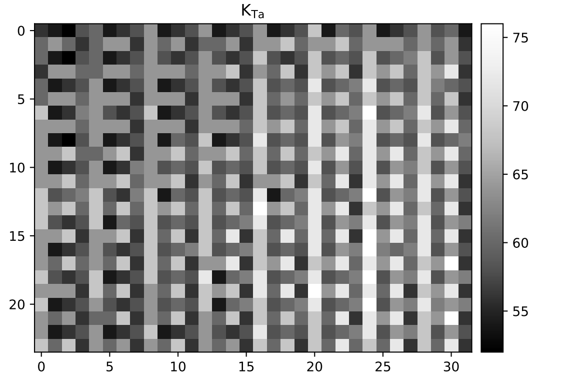

(c) Per-pixel calibration parameter values for parameter K_{Ta} repeat based on a pattern. Because of the representation uncertainty in the calibration data, each pixel has an associated probability distribution.

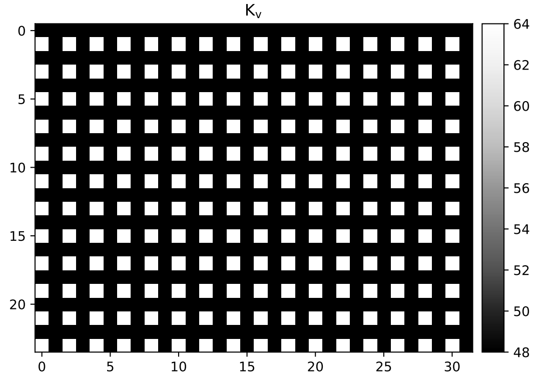

(d) Per-pixel calibration parameter values for parameter K_V take one of two values depending on the pixel location. Because of the representation uncertainty in the calibration data, each of these has an associated probability distribution.

Supplementary Figure 3. Particle values of the per-pixel calibration parameters. Each pixel value has an associated probability distribution that describes the probable values this parameter can take due to representation uncertainty in the calibration data.

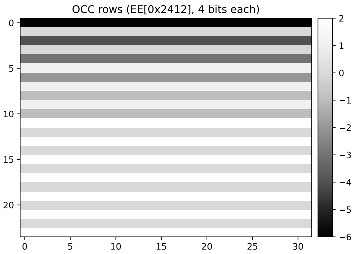

(a) Per-row calibration data for offset calibration parameters.

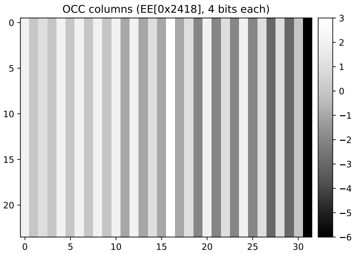

(b) Per-column calibration data for offset calibration parameters.

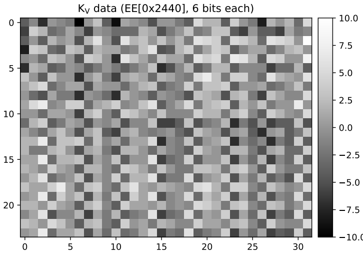

(c) Per-pixel calibration data remainder for offset calibration parameters.

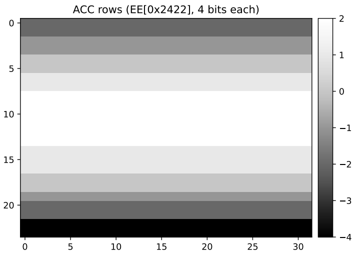

(d) Per-row calibration data for α calibration parameters.

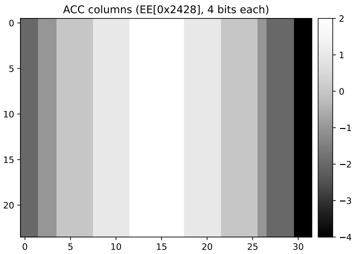

(e) Per-column calibration data for α calibration parameters.

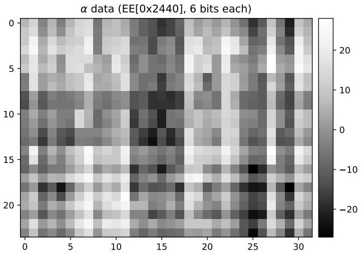

(f) Per-pixel calibration data remainder for α calibration parameters.

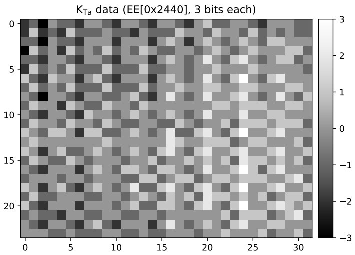

(g) Per-pixel calibration data for K_{Ta} calibration parameters.

Supplementary Figure 4. Pixel-location-dependent calibration data for the MLX90640 measurement conversion routines. Every calibration data integer value X from the sensor EEPROM represents a fundamentally real-valued quantity that could have been anywhere in the range $(X - 0.5, X + 0.5)$ before the calibration process rounded it to the integer X. Because the sensor driver constructs the K_V calibration parameter by selecting between four scalar calibration data values based on the pixel location and scaling the selection, we do not show per-pixel calibration data for K_V here.

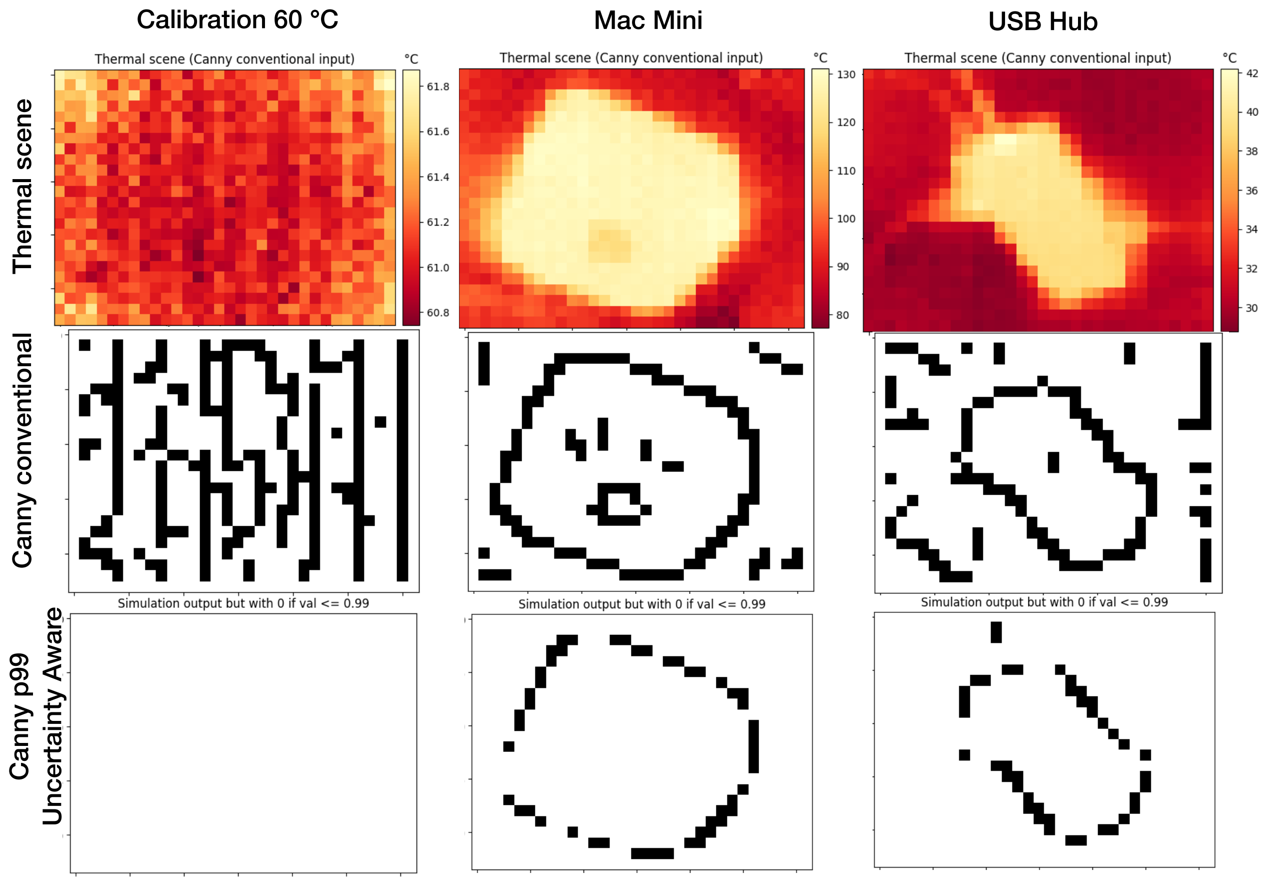

Supplementary Figure 5. Edge detection thermal image output, conventional edge detection, result, and uncertainty-aware edge detection p99 result for the scenes Calibration Source 60 °C, Mac Mini M4, and USB Hub. For all tested scenes the conventional results show artifact that relate to the representation uncertainty of the sensor calibration data. The uncertainty-aware version of the algorithm can better marginalize these artifacts to a cleaner image using a tunable probability threshold. For the p99 threshold, the uncertainty-aware approach achieves 100%, 96%, and 96.8% accuracy, as much as 5.3% higher than the conventional output. For the cases of the Mac Mini M4 and the USB Hub this accuracy increase comes with a drop to sensitivity. Applications can tune the probability threshold to their accuracy, precision, and sensitivity requirements.

Supplementary Table 5. Performance effects of taking temperature-pixel uncertainty into account for edge detection with the Canny filter. Bold typeface is headers.

		Conventional	Uncertainty-Aware		
**Scene**	**Metric**		p80	p90	p99
**Calibration Source 60 °C**	Accuracy	0.7083	1.0000	1.0000	1.0000
	Precision	-	-	-	-
	Sensitivity	-	-	-	-
**Ferrite 50 °C**	Accuracy	0.9779	0.9935	0.9987	1.0000
	Precision	0.7536	0.9123	0.9811	1.0000
	Sensitivity	1.0000	1.0000	1.0000	1.0000
**Heater Plate Corners**	Accuracy	0.9596	0.9701	0.9596	0.9401
	Precision	0.7732	1.0000	1.0000	1.0000
	Sensitivity	0.8929	0.7262	0.6310	0.4524
**Heater Plate Diagonal**	Accuracy	0.9544	0.9531	0.9518	0.9310
	Precision	0.7414	0.8395	0.8857	0.8519
	Sensitivity	0.9451	0.7473	0.6813	0.5055
**Heater Plate Curve**	Accuracy	0.9414	0.9440	0.9271	0.8919
	Precision	0.7623	1.0000	1.0000	1.0000
	Sensitivity	0.8532	0.6055	0.4862	0.2385
**Metal Hob**	Accuracy	0.9362	0.9857	0.9935	0.9948
	Precision	0.3553	0.7353	0.8929	0.9600
	Sensitivity	1.0000	0.9259	0.9259	0.8889
**Face**	Accuracy	0.9049	0.9622	0.9531	0.9518
	Precision	0.4857	0.7941	0.7797	0.8810
	Sensitivity	0.9855	0.7826	0.6667	0.5362
**Thumb Up**	Accuracy	0.8984	0.9740	0.9753	0.9688
	Precision	0.4552	0.9423	0.9796	0.9773
	Sensitivity	0.9242	0.7424	0.7273	0.6515
**Mac Mini M4**	Accuracy	0.9427	0.9700	0.9700	0.9609
	Precision	0.6451	0.8701	0.9130	1.0000
	Sensitivity	1.0000	0.8375	0.7875	0.6250
**USB Hub**	Accuracy	0.9154	0.9831	0.9805	0.9688
	Precision	0.4836	0.9138	0.9259	0.9512
	Sensitivity	0.9672	0.8689	0.8197	0.6393
**(Totals:)**	Accuracy	0.9139	0.9736	0.9710	0.9608
	Precision	0.4910	0.8935	0.9262	0.9543
	Sensitivity	0.9405	0.7746	0.7074	0.5556

The subject is against a non-uniform emissive background in scenes USB Hub, Mac Mini M4, Thumb Up, Face, and Metal Hob.

Supplementary Table 6. Uncertainty statistics for different calibration data storage scenarios. Scenarios that need more memory result to less epistemic uncertainty in the sensor output, with the IEEE-754 32-bit scenario leading to 99.99% smaller error statistics across all datasets. While the IEEE-754 16-bit requires more memory than the TwiceBits scenario, it yields less stable benefit compared to the nearest-integer rounding baseline. Bold typeface is headers.

(Scenario:)	FP16	FP32	TwiceBits	TwoMoreBits
**Metric**	**%Change**			
MinMAE	-76.9172	-99.9980	-93.8324	-75.0021
MeanMAE	-79.2225	-99.9982	-93.8263	-75.0030
MaxMAE	-84.0131	-99.9986	-93.8185	-75.0074
MinMaxAE	-79.4713	-99.9980	-94.1962	-75.2442
MeanMaxAE	-81.9085	-99.9983	-94.2508	-75.3493
MaxMaxAE	-86.2310	-99.9987	-94.3053	-75.6145
MinMRE	-77.1726	-99.9980	-93.8320	-75.0035
MeanMRE	-79.2099	-99.9982	-93.8268	-75.0050
MaxMRE	-83.9950	-99.9986	-93.8211	-75.0106
MinMaxRE	-79.7551	-99.9981	-94.1884	-75.2225
MeanMaxRE	-81.9814	-99.9983	-94.2321	-75.2751
MaxMaxRE	-86.2583	-99.9987	-94.2479	-75.3987
MinStd	-82.9334	-99.9979	-93.8439	-75.0042
MeanStd	-85.0140	-99.9982	-93.8369	-75.0061
MaxStd	-88.5936	-99.9986	-93.8294	-75.0144
MinCI95	-82.7544	-99.9980	-93.8570	-75.0077
MeanCI95	-84.8464	-99.9982	-93.8491	-75.0078
MaxCI95	-88.4642	-99.9986	-93.8415	-75.0184
