# Supplementary material for: Digital methods to quantify sensor output uncertainty in real time
Source: Commun Eng. 2026 May 13;5:135. doi: 10.1038/s44172-026-00679-4 (PMC13396527; doi:10.1038/s44172-026-00679-4)
Supplement: Supplementary file 1 — Transparent Peer Review file [file 44172_2026_679_MOESM1_ESM.pdf]

nature portfolio

Peer Review File

Digital Methods to Quantify Sensor Output Uncertainty in Real Time

Corresponding Author: Mr Orestis Kaparounakis

This file contains all reviewer reports in order by version, followed by all author rebuttals in order by version.

Version 0:

Reviewer comments:

Reviewer #1

(Remarks to the Author)
Dear Authors,

In your article titled “Efficient Digital Methods to Quantify Sensor Output Uncertainty”, you present a detector-characterisation method that enables the quantification of inherent uncertainties in calibration parameters, whether epistemic or aleatoric in nature. You focus on the specific use case of the MLX 90640 detector system, its calibration, and its data-correction requirements.

The publication is well written and clearly summarizes your findings, based on data acquired with the MLX 90640, as well as the conclusions drawn from it. This part of your work is very solid and grounded in sound methodology.

However, you also make several very strong statements that, in my view, do not reflect state-of-the-art design principles applied by detector developers during the design of modern detector systems, regardless of the application or wavelength range (IR, UV, visible, X-ray, or gamma-ray). While some of our findings may indeed be limitations of the MLX 90640, they cannot be generalized to other systems. A few illustrative examples are:

• “… how epistemic uncertainty in the calibration data and the quantization occurring in the signal-processing chain of sensors impacts their accuracy in a way hitherto not recognized in the research literature.”
• “Yet, the inherent uncertainties associated with sensor components remain poorly understood.”
• “Related work has not succeeded in sufficiently addressing the fact that, for fundamental reasons of the nature of sensors, there is unavoidable uncertainty about the sensor outputs, even if we assume a perfect measurement that has zero noise.”
• “Because the representation uncertainty of the calibration parameters causes sensor output uncertainty to be greater than the nominal sensor accuracy of ±3◦C[1], as Section 2 shows, application engineers should not ignore it.”

My own experience in detector system design leads me to a fundamentally different conclusion. To illustrate this, let me consider the well-known quantisation error of an ADC. A general detector design objective is to avoid allowing systematic uncertainties to dominate the overall error budget of a detection system. While constraints such as power consumption or limited memory can sometimes prevent this, a number of established methods exist to minimise quantisation errors, amongst others these are:

• increasing bit depth,
• appliying dithering,
• using oversampling techniques, and
• employing error-feedback corretions.

These methods are well known, well documented, and widely understood. For this reason, I cannot support statements implying that such effects are “not recognized in the research literature” or “not sufficiently addressed.”

Removing the general and somewhat speculative claims from the manuscript results in a paper that essentially summarizes the calibration procedure for a specific IR detector system. The innovation level of the work then appears very limited.

Overall, I cannot support acceptance of this publication in its current form.

Reviewer #2

(Remarks to the Author)
The paper addresses the propagation of uncertainties in sensor systems, focusing on epistemic uncertainty introduced by quantization of calibration constants and sensor outputs. These uncertainties persist even under ideal, noiseless conditions. The authors demonstrate that such uncertainty representation is inherent in calibration-compensated infrared array sensors, varies across pixels and temperatures, and cannot be treated as a fixed offset. A key contribution is the demonstration that real-time uncertainty tracking is feasible on low-power embedded systems using FPGA-based implementations.

Major Claims
==========
1) Epistemic uncertainties arising from calibration and quantization are underestimated in current literature.
2) Real-time assessment of uncertainty improves application-level accuracy, as shown in an edge detection scenario using thermal imaging.
Both claims are supported by experimental evidence, including FPGA-based hardware acceleration for uncertainty tracking.

Novelty
======
The first claim aligns with established metrology principles, notably the Guide to the Expression of Uncertainty in Measurement (GUM, JCGM 100:2008), which emphasizes uncertainty evaluation post-calibration. The paper's novelty lies in highlighting the impact of representation uncertainty within embedded sensor systems and demonstrating real-time uncertainty propagation on FPGA hardware. This practical implementation is, in my opinion, the most novel contribution of the paper.

Strengths
========
- Comprehensive analysis of uncertainty propagation from calibration data to sensor outputs.
- Practical demonstration of real-time uncertainty tracking on resource-constrained hardware.
- (Promised) open dataset and reproducibility of results enhance the paper's value.

Limitations and Suggestions
======================
- The paper's premise feels somewhat indirect: embedded systems have limited memory and computing power, which introduces epistemic uncertainty through ADC resolution and fixed-point computation; adding FPGA-based processing then estimates this uncertainty in real time and improves results. A discussion on whether using floating-point coefficients and higher-precision calibration data could reduce uncertainty at its source would greatly enhance the paper. In other words, is the limitation primarily due to calibration data representation, subsequent calculations, or ADC resolution? Could the proposed framework also help improve calibration procedures?
- Modern industrial sensors often employ high-resolution ADCs (24–32 bit) and floating-point computations. Simulating the proposed approach to such systems would strengthen generalizability.
- The edge detection example, while illustrative, relies on a sensor with inherent limitations (relatively low resolution, emissivity factor uncertainty, ...). Broader applicability to other domains remains unclear.
- Additional experiments could improve the robustness claim: scenes with multiple objects, curved boundaries, and non-uniform emissive backgrounds.
- The discussion could better contextualize the work within related literature on real-time uncertainty quantification, such as Zhou et al.'s recent study on real-time uncertainty quantification for KLT-based displacement estimation.
- The cited maximum relative error (25.7%) appears unusually high. Clarification is needed on how this was calculated. Furthermore, consider that 0 °C should likely not be used as the zero reference for temperature; Kelvin is likely a better choice as it is proportional to the thermal energy. In addition, I assume that the Fluke 4180 setpoint was used as the actual value.
- The paper title could be made more precise e.g., Efficient Methods to Quantify Sensor
Output Uncertainty in Real-Time.

Impact
======
The paper demonstrates that real-time, low-power uncertainty quantification is both feasible and beneficial, potentially influencing future research in embedded thermal imaging with uncertainty-aware decision-making. However, further exploration of diverse applications and higher-end sensor systems would enhance its relevance.

Recommendation
==============
The manuscript presents promising ideas and practical results. With expanded experimental validation, deeper engagement with existing standards & related work, and expanded discussion, it could make a relevant contribution to the field.

Reviewer #3

(Remarks to the Author)
The manuscript addresses a topic of interest to the readership of Communications Engineering. It is generally written well, and the presented concepts have the potential to be extended to other sensor types. The manuscript's verbiage is supported by figures, tables and equations and the authors have also provided supplementary material which may assist other researchers in further developments.
I only found a few small apparent inconsistencies in the manuscript. For example, in the last paragraph of 2.B, the authors refer to R from (2) as being one of the mutual information factors. However, (2) shows R with index i, suggesting is a per-pixel constant. Another apparent discrepancy is in section 4, where the performances of UxHw-FPGA-5k and EqMCp90-FPGA-5k are compared. the first one is mentioned to complete the per-pixel task in under 4740 ms (4.74 s), whereas the latter one needing at least 203509 ms (203.509 s) indicating a 42.9 speedup. Given the sentence structure, it seems to suggest that the latter achieves a speedup whereas the opposite seems to be true, given the numbers.

Version 1:

Reviewer comments:

Reviewer #1

(Remarks to the Author)
Dear authors,

Thank you very much for revising your manuscript and taking all my comments and suggestions into account. The article now focuses on real-time uncertainty quantification using a thermal sensor as an exemplary show case. The article now carefully addresses existing quantisation-mitigation methods and establishes the relationship between these and the presented method. The manuscript clearly describes the innovative nature of your approach.

I only have a two minor comments on the revised manuscript:

1. Lines 97–98

The authors refer to Figure 1.H, 'Calibration Phase', when discussing the storage of calibration coefficients in memory. Would it make more sense to refer to Figure 1.L, 'Calibration Data Memory', instead?

2. Lines 748–749

I suggest changing the wording of the sentence 'Ideal materials that emit the maximum theoretical radiation are called black bodies'. For clarity, I suggest changing it slightly to 'An ideal object that emits the maximum possible thermal radiation at a given temperature is called a black body.'

Apart from these minor modifications, I recommend your manuscript for publication. In my opinion, it does not need to be reviewed again.

Yours sincerely

Reviewer #2

(Remarks to the Author)
The paper "Efficient Methods to Quantify Sensor Output Uncertainty in Real-Time" addresses three main contributions:
1) Quantification of epistemic uncertainty from finite-precision calibration data storage in IR array sensors.
2) A practical FPGA-based framework for real-time uncertainty quantification (40-100x speedups over Monte Carlo).
3) Demonstration that real-time uncertainty quantification improves downstream applications (edge detection) and informs sensor design trade-offs.

Compared to the original manuscript, the authors have significantly improved the paper. The context and contributions are now clearer. They have shown the effects of reducing uncertainty by adding "more bits" to calibration coefficients. The edge detection example is now more convincing with diverse test scenes.

The strengths of the paper after this revision are:
* Clear scope: representation uncertainty from calibration data quantization
* Comprehensive experimental validation with rigorous metrics (Wasserstein distance, speedups, power consumption)
* Practical demonstration on resource-constrained hardware
* Design trade-off analysis valuable for sensor designers
* Deterministic approach enables reproducible, efficient propagation

The authors have successfully addressed my first-round of concerns. I am only missing a brief justification for temperature reference choice in Methods section, which is only a minor concern.

Open Access This Peer Review File is licensed under a Creative Commons Attribution 4.0 International License, which permits use, sharing, adaptation, distribution and reproduction in any medium or format, as long as you give appropriate credit to the original author(s) and the source, provide a link to the Creative Commons license, and indicate if changes were made.
In cases where reviewers are anonymous, credit should be given to 'Anonymous Referee' and the source.
The images or other third party material in this Peer Review File are included in the article's Creative Commons license, unless indicated otherwise in a credit line to the material. If material is not included in the article's Creative Commons license and your intended use is not permitted by statutory regulation or exceeds the permitted use, you will need to obtain permission directly from the copyright holder.
To view a copy of this license, visit https://creativecommons.org/licenses/by/4.0/

March 5, 2026

Responses to Reviewer Comments for COMMSENG-25-0611-T

Dear Editor and Reviewers,

Thank you for your careful review and for the constructive comments. The feedback helped us clarify the positioning of the manuscript, improve technical precision, and better support the discussion and presentation of the proposed method and its general applicability. This letter addresses the reviewer comments in detail. We have uploaded the revised manuscript which addresses the comments and also the tracked-changes version where removals and additions appear in crossed-out red text and blue text, respectively.

Please find below each reviewer comment in italics, followed by our response. Line number ranges refer to the tracked-changes version of the manuscript.

Response to Reviewer 1

Comment 1

Remove strong statements to better reflect contemporary design principles in detector development.

Response

We have revised the manuscript to ensure that it does not claim that detector developers overlook quantization or nor that the effect is new (lines 101–111). The revised manuscript focuses on one mechanism, i.e., finite-precision storage of calibration data, and quantifies its impact for a concrete sensor and conversion routine. We have restricted the manuscript discussion to the specific mechanism we study while drawing connections to other mechanisms for better contextualization ("Representation uncertainty" paragraph, lines 164–178, and Supplementary Section SM.B). Overall, we have increased the focus of the manuscript on the hardware-based acceleration approach for dynamic online uncertainty quantification and have brought the relevant section forward to be Section 1. The revised contributions list now better communicates this (line 116).

Comment 2

Support the general applicability of the method beyond the demonstrated sensor system.

Response

We have increased the focus of the hardware-based accelerated approach for dynamic online uncertainty quantification wherein we use the specific infrared sensor as a motivating and driving example. The revised manuscript uses the thermal sensor as a detailed case study (Section 2.A) and presents the on-device uncertainty quantification framework as the main contribution, which other devices can adapt when their calibration representation and compensation code match the same pattern. The revised manuscript clarifies that the proposed method applies when a device stores calibration data in finite-precision memory, reconstructs calibration parameters at runtime, and runs a deterministic conversion routine that reuses those parameters. These clarifications appear on the blue text in lines 95–100, the introductory "Representation

uncertainty" paragraph (lines 164–178), Section 2 lines 231–235, and Section 2.B lines 308–326.

Comment 3
Clarify connections to established quantization-error mitigation methods.

Response
The revised manuscript improves the positioning with regards to established quantization-mitigation methods. Dithering, oversampling, and error feedback target time-varying quantization; they do not restore information in once-quantized stored coefficients. The revised manuscript clarifies this in the "Representation uncertainty" paragraph of the introductory section (blue text, lines 164–178) and, in greater detail, in Supplementary Material Section SM.B under "Relation to quantization-mitigation methods" (blue text, lines 838–856).

We have appropriately removed ambiguous wordings to ensure that the manuscript does not argue that other uncertainty sources do not matter, and does not propose a new mitigation technique. The manuscript instead quantifies the output uncertainty implied by a given calibration encoding and compensation implementation, and the presented framework supports in design trade-offs such as selecting coefficient bit depth, memory budget, and representation format.

Comment 4
Motivate the innovation level of the work after moderating general claims.

Response
The contribution is a practical on-device method to propagate calibration-data representation uncertainty through real conversion code in real time, with measured iso-quality 42.9x and 94.4x speedups versus Monte Carlo on two low-power FPGA platforms. The revised manuscript more clearly focuses on this contribution by prioritizing the discussion of the hardware-based accelerated approach for the dynamic online uncertainty quantification (lines 116–117), wherein the specific infrared sensor serves as a motivating and driving example (lines 125–126). We have revised the manuscript to move this discussion to Section 1 to improve understanding while also keeping an abridged version of the relevant context (previous Section 1) as the "Representation uncertainty" paragraph (lines 164–178), with the rest of the context now in Supplementary Section SM.B.

To provide additional evidence of usefulness beyond runtime gains, the revised manuscript includes two experimental proof-of-usefulness examples: the edge detection application that uses the quantified uncertainty to tune edge classification performance (Section 3.A), and a new sensor-design application that uses the method to compare four calibration data storage scenarios and their memory–uncertainty trade-offs for faster design feedback (Section 3.B, lines 401–454). The revised contributions list reflects both applications (lines 143–148).

Response to Reviewer 2

Comment 1
Clarify the presentation of the premise and the proposed solution which feels somewhat indirect.

Response
The revised manuscript introduces the proposed on-device uncertainty quantification method first, and it then uses the thermal sensor as a case study to ground the discussion. Section 1 now presents the uncertainty quantification framework while the

mechanism of representation uncertainty in a focused paragraph immediately before it (lines 164–178), which addresses the ambiguity that the manuscript motivates the problem indirectly through the case study. Section 2 then applies the framework to the infrared sensor as a detailed case study.

Comment 2
Clarify the source of epistemic uncertainty in the examined setting, and show how coefficient formats (and memory budgets) change it.

Response
This work studies epistemic uncertainty that arises when a sensor stores its calibration coefficients in finite-precision on-device memory, and it later reconstructs and uses those coefficients in the compensation and conversion routine. The uncertainty therefore comes primarily from information loss during coefficient encoding and decoding in this case study, rather than from ADC quantization in the measurement path. The revision clarifies that ADC resolution and fixed-point arithmetic can also introduce epistemic effects in other settings and that our work focuses specifically on coefficient-storage quantization so the mechanism, and its impact, remain identifiable and measurable (lines 93–100).

The data storage format determines both the implied representation uncertainty and the required memory budget. We have extended the work with the new Section 3.B which adds reviewer-proposed experiments and compares four coefficient-storage strategies against the baseline, reporting the resulting uncertainty changes alongside the on-board memory requirements. This added validation supports a design-space exploration use case during the calibration process, where our proposed method accelerates trade-offs between coefficient precision, memory budget, and the resulting output uncertainty. The revised contributions list reflects this addition (lines 146–148).

Comment 3
Improve motivation for the edge detection application and justify broader applicability.

Response
The revised manuscript clarifies that the edge detection section serves as a proof-of-usefulness application: it shows how the quantified output uncertainty from calibration-coefficient representation affects downstream decisions, rather than presenting a new edge detection method (lines 143–145, 361–365, and 507–514). The revision also expands the evaluation to address robustness concerns (lines 389–400). Section 3.A and Supplementary Material Section SM.D now report results over ten thermal scenes that include harder conditions, including multiple objects in the field of view, curved and irregular boundaries, and non-uniform emissive backgrounds. The new Supplementary Figure SM.F11 contains visuals for three of the ten scenes. The revised text summarizes how uncertainty-aware processing changes decision outcomes and it reports the resulting trade-offs in accuracy, precision, and sensitivity, with the per-scene breakdown provided in Supplementary Material SM.D, lines 993–1014.

Comment 4
Address generalizability to modern industrial sensors that use high-resolution ADCs and floating-point computations.

Response
Higher-resolution ADCs and floating-point arithmetic reduce several quantization and rounding effects in the measurement and compute path. The mechanism we study still arises whenever a device stores real-valued calibration parameters as finite-precision calibration data in non-volatile memory and later reconstructs and reuses them in the conversion routine, which remains common even in higher-end sensors due to storage, bandwidth, or legacy interface constraints. The revised manuscript clarifies these points in lines 93–100, 164–178, 308–326, and 813–827.

Even when a system stores calibration data in floating-point format, the storage precision still bounds the recoverable information and therefore bounds the residual representation uncertainty. The new Section 3.B performs and discusses the reviewer-proposed experiments quantifies this trend directly by examining four coefficient-storage formats and bit widths, which shows how increasing stored precision shrinks the propagated output uncertainty at a corresponding memory cost. The new Figure 6 shows the effective difference in uncertainty for the different calibration data storage strategies.

Comment 5

Suggestion on reference to expand the discussion with context for uncertainty quantification for KLT-based displacement estimation.

Response

We added a citation and a brief qualitative comparison with bootstrap-based real-time uncertainty quantification (as in KLT-based displacement estimation) in Section 1 lines 186 and 199–206. The revision clarifies how bootstrap-based approaches quantify uncertainty by resampling a per-input ensemble of conventional pipeline outputs to compute uncertainty statistics (e.g., confidence intervals) around an estimate. In contrast, this work targets parametric epistemic uncertainty induced by finite-precision storage of calibration data and uses the UxHw system to propagate that representation uncertainty through calibration-extraction and calibration-compensation code, which yields an approximate uncertainty distribution for each calibration-compensated sensor output.

Comment 6

Clarify calculation of maximum relative error and the baseline.

Response

The revised manuscript clarifies the definition of the maximum relative error along all metrics in the Methods in Section 5, lines 571–584, specifically Equation 7. For the metrics in Equations 4–7, the sensor's conventional output provides the single point value under evaluation, and the distribution of probable outputs implied by the calibration representation uncertainty provides the reference used to assess error.

The evaluation computes the relative metrics directly on the explicit outputs of the sensor's conversion routines, without any post-processing transformations such as unit changes, to match how the device is used in the field and to keep the reported relative errors tied to the sensor's native output scale.

Comment 7

Change the manuscript title to "Efficient Methods to Quantify Sensor Output Uncertainty in Real-Time" to make it more precise.

Response

We thank the reviewer for the suggestion to clarify the manuscript title and more precisely communicate the enclosed content. We adopted the suggested title.

Response to Reviewer 3

Comment 1

Clarify the notation in Equation 2 and the discussion of mutual-information factors.

Response

The revised manuscript clarifies the role of R and C in Equation 2 by using explicit row and column subscripts for the relevant symbols, which removes the earlier ambiguity about whether R is per-pixel or shared across pixels. The discussion now states explicitly that the shared information in R between a_3 and a_4 arises because the third and fourth pixels lie on the same row. To improve flow and keep the main text focused on the proposed technique, the correlation example around a_3 and a_4 now appears in Supplementary Section SM.D (lines 982–984).

Comment 2

Correct the attribution of the reported speedup in the runtime comparison.

Response

We corrected the sentence in the runtime comparison to attribute the 42.9x speedup to UxHw-FPGA-5k relative to the corresponding EqMCp90-FPGA-5k baseline. We did the same clarification for the UxHw-FPGA-17k results. The revised text now names both methods explicitly and states the direction of the comparison unambiguously (lines 346–347 and 349–350).

Summary of Changes

1. The revision clarifies the positioning of our manuscript to foreground the proposed technique for real-time uncertainty quantification (Section 1) and leverage the thermal sensor as a detailed case study (Section 2). The revision improves understandability of the manuscript by condensing the old Section 1 in the last paragraph of the introduction. The old Section 1 is now in Supplementary Material Section SM.B.
2. The revision clarifies the claims to the specific uncertainty mechanism that we study (calibration-data representation uncertainty), and adds positioning relative to existing quantization-mitigation methods. The revision also better communicates the generalizability and applicability of our approach for other sensors and systems.
3. The revision expands evaluation with ten edge-detection scenes and reports accuracy, precision, and sensitivity trade-offs (Section 3.A).
4. The revision expands the evaluation to examine storage-format and memory-budget scenarios (Section 3.B) and quantifies how increased coefficient precision reduces propagated uncertainty.

We believe these revisions address all concerns and improve the manuscript as a whole. Thank you for your time and consideration.

Yours sincerely,
the authors

April 2, 2026

Responses to Reviewer Comments for COMMSENG-25-0611A

Dear Editor and Reviewers,

Thank you for your review and the constructive comments. The comments helped us polish final details of the manuscript.

Please find below each reviewer comment in italics, followed by our response.

Response to Reviewer 1

Comment 1
Refer to correct part of illustration in introduction.

Response
We have revised the text to correctly point to Figure 1.L according to the reviewer suggestion.

Comment 2
Improve clarity of ideal black bodies statement.

Response
We have amended the wording of ideal black-body radiation according to the reviewer suggestion.

Response to Reviewer 2

Comment 1
Justify the temperature reference choice in Methods section.

Response
The revised manuscript justifies the choice for the temperature reference in the Metrics paragraph of Section 6.B (Methods). The chosen reference corresponds to the probable true temperature value so relative metrics use this as the true-value reference.

We believe these changes address the comments of this revision. We have also adapted the manuscript to comply with the journal policies. Thank you for your time and consideration.

Yours sincerely,
the authors
